# Inhibition of Reaction Layer Formation on MgO(100) by Doping with Trace Amounts of Iron

**DOI:** 10.1021/acs.jpcc.4c06311

**Published:** 2025-02-12

**Authors:** Gabriela Camacho Meneses, Juliane Weber, Raphaël P. Hermann, Anna Wanhala, Joanne E. Stubbs, Peter J. Eng, Ke Yuan, Albina Y. Borisevich, Matthew G. Boebinger, Tingting Liu, Andrew G. Stack, Jacquelyn N. Bracco

**Affiliations:** † School of Earth and Environmental Sciences, Queens College, City University of New York, New York Queens 11367-0904, United States; ‡ Chemical Sciences Division, Oak Ridge National Laboratory, Oak Ridge, Tennessee 37831, United States; § Materials Science and Technology Division, Oak Ridge National Laboratory, Oak Ridge, Tennessee 37831, United States; ∥ Center for Advanced Radiation Sources, The University of Chicago, Chicago, Illinois 60637, United States; ⊥ James Franck Institute, The University of Chicago, Chicago, Illinois 60637, United States; # Center for Nanophase Materials Sciences, Oak Ridge National Laboratory, Oak Ridge, Tennessee 37831, United States; ∇ Earth and Environmental Sciences, Graduate Center, City, University of New York, New York, New York 10016-4309, United States

## Abstract

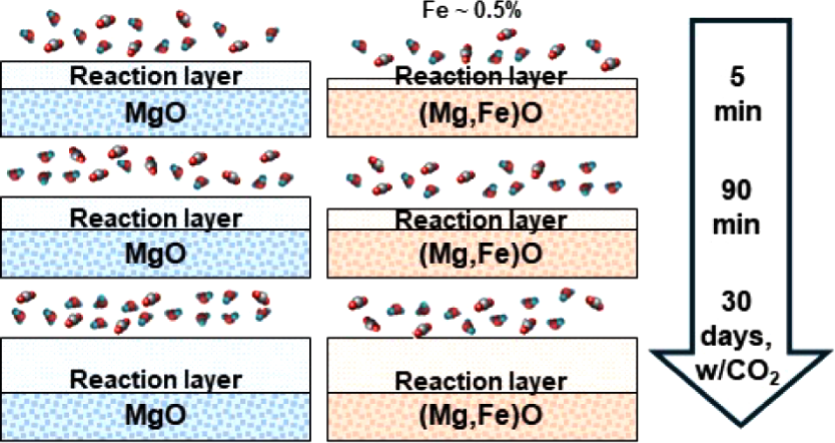

Despite extensive research on MgO’s reactivity in the presence of CO_2_ under various conditions, little is known about whether impurities incorporated into the solid, such as iron, enhance or impede hydroxylation and carbonation reactions. The purity of the MgO required for the successful implementation of MgO looping as a direct air capture technology affects the deployment costs. With this motivation, we tested how incorporated iron impacts MgO (100) reactivity and passivation layer formation under ambient conditions by using atomic force microscopy, electron microscopy, and synchrotron-based X-ray scattering. Based on electron microprobe analysis, our MgO samples were 0.5 wt % iron, and Mössbauer spectroscopy results indicated that 70% of the iron is present as Fe^(II)^. We find that even these low levels of iron dopants impeded both the hydroxylation at various relative humidities (10%, 33%, 75%, and >95%) and carbonation in CO_2_ (33%, 75%, and >95%) on the (100) surface. Crystalline reaction products were formed. Reaction layers on the sample were easily removed by exposing the sample to deionized water for 2 min. Overall, our findings demonstrate that the presence of iron dopants slows the reaction rate of MgO, indicating that MgO without incorporated iron is preferable for mineral looping applications.

## Introduction

Negative emissions strategies will be necessary to remove CO_2_ from the atmosphere and constrain global temperature rise to 1.5–2 °C. One potential technology class is direct air capture (DAC) technology, in which a solid or liquid is used to absorb or remove CO_2_ directly from the atmosphere for storage or utilization.^1^ A promising DAC technique is the mineralization of alkaline metal oxide minerals, such as MgO or CaO, using a mineral looping process.^2^ In this process, the metal oxide reacts with CO_2_ in the atmosphere over a specified time to form carbonate minerals, which are often hydrated metal carbonates.^2−5^ The reacted alkaline earth oxide is then calcined to separate CO_2_ for capture, which may subsequently be sequestered or utilized to produce bioplastics,^6^ cleaner concrete,^7^ and green fuels.^8−10^ This approach can potentially be scaled economically to gigaton levels (2–3 Gt/year) to help meet U.S. climate targets.^2^ MgO has a lower calcination temperature as compared with CaO, which may make it more favorable for DAC due to the lower energy requirements.^2^ Numerous recent studies have analyzed the hydroxylation of MgO,^3,4,11−14^ though the majority of these used pure MgO with limited impurities present. However, impurities are expected to be present in alkaline earth oxides produced from natural sources (such as iron, a common impurity in feedstock magnesite and dolomite for MgO production and limestone for CaO production), as well as MgO produced industrially from brines (such as calcium, a common component of seawater).^15,16^

Use of natural materials as feedstocks for MgO requires the calcining of magnesite or dolomite to create the initial MgO used for the looping process. The magnesite or dolomite that is being calcined can be either mined or precipitated from brines. To extract 1 Gt CO_2_/yr from the atmosphere would require around 1.9 Gt magnesite to produce the needed MgO based on mining, which is approximately 25% of the projected global magnesite reserves.^17^ For source material that will be economically advantageous, both magnesite and dolomite are likely to have impurities present, particularly divalent cations such as Fe^2+^ that can substitute for Mg in the MgCO_3_ or CaMg(CO_3_)_2_ crystal structure.^18^ Magnesite deposits have a wide range of iron contents depending on the type of deposit. Sparry (or macrocrystalline) deposits can commonly have iron oxide content from 1 to 8%, while cryptocrystalline deposits typically have iron oxide content below 1%.^19^ For example, deposits in Central Brazil^20^ and British Columbia, Canada,^21^ have ranges of iron oxide content of approximately 0.5–1.5%. MgO looping with impurities such as iron could either enhance or impede various parts of the hydroxylation, carbonation, and film growth process, but the specifics and mechanisms remain unknown under the conditions relevant to DAC. This is a concern since, when adopting this approach, additional expenses and logistics must be assessed to establish whether pure MgO is required for the success of this DAC technology.

Previous research on the impact of iron impurities on MgO properties primarily focused on the impact of iron on mechanical properties and behavior but not on its reactivity.^22−24^ For example, implantation of iron into nonmagnetic MgO could induce magnetism.^25^ Iron implantation using an ion beam accelerator led to a weakening of the coordination of the magnesium in the MgO and the presence of iron with an average oxidation state of 2.3, potentially due to the presence of both Fe(II) and Fe(III).^25^ Iron can be introduced into MgO as either a substitution for magnesium as Fe^2+^ or Fe^3+^, precipitation as a separate iron-bearing phase, or as metallic iron, but MgO hardening is most extensive when the iron is present as phase-segregated precipitates (MgO.Fe_2_O_3_).^23^ The oxidation state of iron also impacts physical properties; for example, Fe^3+^ causes more hardening of MgO than Fe^2+^ due to more similarities in size as compared with Mg^2+^.^22^ Iron oxides in solid solution in MgO also seem to slow the early stages of hydroxylation reactions due to the impacts of Fe^2+^ and Fe^3+^ on the MgO lattice, though the general hydroxylation mechanism appears unchanged.^26^ In a recent study using metadynamics, iron inhibited MgO carbonation, likely by leaching from the surface and neutralizing some of the basicity of the near-surface layer.^27^ While these studies demonstrate that substitution of magnesium for iron and the formation of separate iron phases can affect the physical and electromagnetic properties of MgO, less is understood about how iron impurities affect MgO reactivity.

Iron is known to decrease the reactivity of mineral phases and lead to the formation of passivation layers in silicate mineral systems, such as chrysotile mining residues during carbonation^28^ and olivine during carbonation in the presence of wet CO_2_.^29,30^ In addition, two recent studies found that impurities affect the reactivity of MgO and Mg(OH)_2_ (brucite).^31,32^ For example, the presence of CaO in MgO shifts the pH of water in contact with MgO from ∼10.5 to 12.8, which in turn causes changes in the resulting brucite morphology.^32^ A recent study shows that Fe(II) present in brucite decreases carbonation efficiency in both oxic and anoxic conditions, and the effect increases with increasing iron incorporation in the brucite.^31^ The effect is less severe in anoxic conditions since Fe(II) can incorporate into stable carbonates, such as siderite. Oxidation of Fe(II) can also create an acid that could dissolve Mg-carbonate phases. Finally, iron impurities not only incorporate into MgO but can also be present in surrounding waters during the hydroxylation reaction. In another recent study, we found the presence of Fe(II) during the hydroxylation of MgO nanocubes (predominantly (100) surfaces) leads to the formation of nanoscale Fe-oxides, which increase its carbonation.^33^

MgO(100) surfaces have low surface polarity, which is thought to make them more stable in air than the (111) or (110).^34^ The (100) also has perfect cleavage, which makes it well-suited for experiments. MgO(100) is composed of alternating magnesium and oxygen atoms, but the (111) would be expected to be composed of either magnesium or oxygen atoms, which makes it prone to reconstruction due to its polar nature and low stability.^35^ While the (100) is nonpolar and more stable in air, water can still dissociate and adsorb on the MgO(100) surface, even at low defect densities.^36^ Upon hydroxylation, the (100) may then restructure into (111) nanofacets that are similar to brucite (001) surfaces.^37^ However, while impurities can affect brucite reactivity, it is not clear that brucite is always formed as an intermediate in the carbonation reaction of MgO as compared with hydroxylation. Therefore, the role of incorporated iron in the initial stages of MgO hydroxylation and carbonation needs to be understood to assess the potential of mineral looping in a more realistic scenario.

An understanding of how impurities such as iron affect hydroxylation, carbonation, and film growth on MgO surfaces is critical for a successful DAC strategy using mineral looping. To resolve the impact of iron impurities on MgO, we report X-ray scattering and microscopy measurements designed to investigate how iron impurities affect hydroxylation, carbonation, and film growth on the MgO(100) surface.

## Methods

### Materials

At Oak Ridge National Laboratory, high-purity, microbubble-free (Mg,Fe)O single crystals with a size of 2–3 cm were previously synthesized utilizing the carbon arc-fusion method.^38^ Due to passivation of the MgO samples after synthesis,^4^ all samples were cleaved to expose a fresh surface prior to X-ray scattering and microscopy measurements. (Mg,Fe)O samples were characterized to quantify iron concentration using the Cameca SX100 microprobe at the University of Tennessee using a 15 kV/10 nA beam with a 5 μm spot. Standards utilized were MgO for Mg, hematite (Fe_2_O_3_) for Fe, and diopside (CaMgSi_2_O_6_) for Ca. Peak count times were 20 s for Mg and 60 s for Ca and Fe. The estimated three-sigma detection limit was 350 ppm for Mg, 160 ppm for Ca, and 210 ppm for Fe. Backgrounds were measured on both sides of the peak for half peak time on every analysis point.

(Mg,Fe)O single crystal samples showed pieces that were yellow and red in color (Figure S1). Mössbauer spectra on (Mg,Fe)O were obtained from ground pieces of “yellow” and “red” MgO to identify differences in iron oxidation. For low iron concentrations, the ideal thickness for this material is ∼350–400 mg/cm^2^, and we initially weighed and ground pieces close to that mass. For the “red” sample, this turned out to give too much signal (25% absorption), and we removed half of the mass. Spectra were acquired at ambient conditions using a Wissel GmbH constant acceleration drive in the ±4 mm/s velocity range and a Kr-gas proportional counter. Spectra were acquired under the exact same conditions and geometries to allow for quantitative comparison of the spectral area. Alpha-iron foil was used for calibration and serves as an isomer shift reference; note that literature values of isomer shifts have been corrected for the different reference materials here. Since Mössbauer spectroscopy showed differences in the oxidation state of “yellow” and “red” (Mg,Fe)O, we conducted all our experiments using the “yellow” (Mg,Fe)O only.

### In Situ Atomic Force Microscopy

An Asylum Research MFP-3D instrument in droplet setup was used for the in situ AFM experiments. (Mg,Fe)O crystals were cleaved using a razor blade just before the experiment, exposing a fresh mineral surface. PNP-TR-50 AFM tips were used with a resonance frequency of 17 kHz and a force constant of 0.08 N/m, and imaging was done in contact mode. The applied solution was a saturated MgO solution with an adjusted pH of 12.46. The MgO saturated solution was prepared by letting powdered, high-purity MgO react with deionized water for several weeks. The pH was adjusted using NaOH. Prior to the AFM experiment, the solution was filtered using a 0.20 μm filter. No effort was made to exclude or remove dissolved gases from air from the solutions.

### Sample Preparation for Transmission Electron Microscopy and X-Ray Scattering Experiments

Samples for our experiments were reacted either at the X-ray scattering beamline while mounted on the diffractometer (referred to here as in situ) or in a desiccator for a set period of time and then characterized at the beamline (referred to here as ex situ). Table S1 has specifics on the reaction conditions. The in situ samples were cleaved to expose the (100) surface in a pop-up glovebag or glovebox filled with dry N_2_ gas before being taken to the beamline, after which they were reacted for 5–90 min in either humidified N_2_ or CO_2_. The gases were humidified by bubbling them through deionized water in two gas washing bottles in line with each other. No attempt was made to remove oxygen from any of the gases used since the presence of oxygen is expected for DAC conditions. A subset of these samples was then reacted with deionized water to determine whether the film could be removed. Relative humidity (RH) for the dry N_2_ was measured using a hand-held hygrometer and found to be 11–12%; these conditions were labeled as “dry.″ The RH for the humidified N_2_ and CO_2_ was >95%. The ex situ samples were reacted for 8 or 30 days in a desiccator in either air or CO_2_ (1 bar) at 33% or 75% RH. Saturated solutions of NaCl and MgCl_2_ were utilized to regulate the desiccator’s humidity.

### Transmission Electron Microscopy

Transmission electron microscopy (TEM) and scanning transmission electron microscopy (STEM) measurements were conducted using an FEI Titan (60–300 kV) aberration-corrected scanning/transmission electron microscope (S/TEM) at 300 kV. STEM electron energy loss spectroscopy (EELS) measurements were carried out using a Gatan Quantum EEL spectrometer with a dispersion level of 0.3 eV/channel for datasets measured on the (Mg,Fe)O reacted overnight at 11% RH (sample name: (Mg,Fe)O 11p-2) and 0.5 eV/channel for datasets measured on the (Mg,Fe)O reacted at 11% RH for 4 h and a total of 15 min at >95% N_2_ (sample name: (Mg,Fe)O 11p-4h), respectively. The EELS spectrum images are 2D scans over the area, and the respective image is the high angle annular dark field signal acquired concurrently to establish pixel-by-pixel registration of spectral response to image features.

### X-Ray Reflectivity

At the Advanced Photon Source (APS), low-angle X-ray reflectivity (XRR) measurements were conducted at beamline 13-ID-C to evaluate the roughness, density, and thickness of reaction layers of (Mg,Fe)O (100) crystals (lattice parameter = 4.21 Å; unit cell area = 17.72 Å^2^). To avoid the formation of radicals, all measurements for the ex situ and in situ samples were taken in flowing, nominally dry N_2_. A Newport Kappa Six (4 samples + 2 detectors) circle diffractometer and a Pilatus 100 K pixel array detector (Dectris, Inc.) placed 1.1 m from the center of rotation were used for the XRR measurements. Two 1 m long Si mirrors in Kirkpatrick-Baez geometry were used to collimate the beam, and slits measuring 15 × 500 μm were used to define its size. The incident and reflected beam vectors defined the horizontal plane of scattering, and the longer dimension of the beam cross-section was directed perpendicular to this plane. At a given energy (10 keV; λ = 1.24 Å), the XRR intensity was measured as a function of momentum transfer, *Q* = 4π sin(α_i_)/λ, where λ is the X-ray wavelength and α_i_ is the incidence angle with respect to the surface. The XRR for the samples was measured to a maximum 2θ of 12.8°; though depending on the sample quality, some samples were only measured up to a maximum 2θ of 4.8° or 6.8°.

All samples were mounted on the diffractometer within 1 or 2 h after cleaving and stored under dry nitrogen until mounting. The in situ samples were reacted in a humid environment (>95% RH) at the beamline by bubbling either N_2_ or CO_2_ through water in a line, referred to here as humid N_2_ or humid CO_2_, respectively. The reaction times in humid N_2_ were a total of 5, 10, and 15 min, while the reaction times in humid CO_2_ were a total of 5, 10, 15, 20, 30, 60, and 90 min. Post 90 min of reaction with CO_2_, the samples were reacted with deionized water for 2 min before rereacting with humid CO_2_ (see Table S1 for full measurement details). Prior to measuring the sample’s XRR, the sample was exposed to humid N_2_ or CO_2_ for a set amount of time, followed by 10 min of nominally dry N_2_ to completely dry the sample cell and gas lines. The ex situ samples were shipped in bags containing N_2_ gas (less than 25% relative humidity at ambient temperature) to the APS and measured to a 2θ of 12.8° or 8.8° at 33% and 75% RH. To maintain a regulated environment (∼3% RH) at the beamline, the samples were mounted on a 6-circle diffractometer and covered with a Kapton dome under N_2_ flow.

Before fitting, the XRR signals from the detector images were background subtracted and integrated by using a custom MATLAB code. In GenX (ver. 3.6.3), models were fit to the XRR data as a function of 2θ. A total of 4–7 fit parameters were used for each XRR profile. The models consisted of an MgO substrate with variable roughness and one or two thin film layers, of which the density, thickness, and roughness were permitted to vary. Adding more layers produced nonunique solutions and did not significantly enhance the quality of the fits. Additionally, the fit quality did not increase when one of the thin film layers’ densities was fixed to that of Mg(OH)_2_. The following equation was used in the first fits to minimize χ^2^, weighted by the error bars, by using a differential evolution technique.
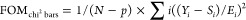


In this case, *N* indicates the total number of data points, *p* indicates the number of free parameters in the fit, *Y_i_* denotes the XRR data, *S_i_* represents the model fit, and *E_i_* represents the error bars. This technique prevents trapping at local minima and allows for a thorough search of the parameter space. From the final fits, we estimated the parameter error bars using the GenX bumps package.^39^

### GIXRD

Grazing incidence X-ray diffraction (GIXRD) scans were collected using the same method as Bracco et al., 2024.^11^ Scans were collected at 10 keV from 2θ = 5–64° using a fixed incidence angle of 0.18°, which is below the critical angle for total external reflection (0.22°) to limit the penetration depth. At this energy, the penetration depth into MgO is 4.4 nm if there is no film present. The center of the detector was scanned in the vertical plane and fixed at 2° in the horizontal (reflecting) plane, and a region of interest was defined with a size of 34 mm × 2.6 mm (horizontal × vertical). Raw intensities were then summed over this ROI with no background subtraction.

## Results and Discussion

### Starting Material Characterization: Electron Microprobe Analysis

Electron Microprobe Analysis (EPMA) shows an average detected dopant level of 0.50 ± 0.04 wt % Fe and 0.006 ± 0.004 wt % Ca (Table S2). Maximum detected concentrations are 0.56 ± 0.04 wt % for Fe and 0.019 ± 0.004 wt % for Ca, respectively. The minimum detected concentration was 0.41 ± 0.04 wt % for Fe and below the detection limit (0.016 wt %) for Ca. There are no contrast variations in the BSE images (Figure S2), which indicates a homogeneous distribution of iron within MgO.

### Starting Material Characterization: Mössbauer Spectroscopy

Mössbauer spectra of (Mg,Fe)O (Figure S3) exhibit a majority single line component centered at ∼1 mm/s, characteristic of Fe(II), with a shoulder at ∼0 mm/s corresponding to an Fe(III) doublet. The data were fitted with one doublet component for Fe(III) and a single and doublet component constrained to the same isomer shift for Fe(II). Spectral parameters are reported in Table S3. Considering the relative amount of material in the samples and the total spectral area, the “red” sample contains 4.7 times more iron than the “yellow” sample. Neglecting possible differences in Lamb-Mössbauer factors for these samples and alpha-iron and comparing to the calibration iron foil, the “yellow” sample contains 0.7(2) wt % iron, whereas the “red” sample contains 3.3(9) wt % of iron.

Prior research on the (Mg,Fe)O system used Mössbauer spectroscopy to determine the local environment of iron,^40^ lattice distortions,^41^ solubility of Fe(II) and Fe(III),^42^ and the position of the iron ions.^43^ When single crystal MgO was doped with Fe(II) at low concentration (1.5 wt %), a single line was observed, with an isomer shift of 1.07(5) mm/s; at higher concentration (18.3 wt % Fe(II), 0.19 wt % Fe(III)), an isomer shift of 1.087(5) mm/s and a quadrupole splitting of 0.630(5) was observed.^41^ The Fe(II) ions can substitute Mg in an octahedral lattice site and primarily yield a singlet line, whereas Fe(III), when substituting Mg creates an Mg^2+^ vacancy, which causes distortion of the octahedral lattice site and yields larger quadrupole splitting on the Fe(III)^43^ as we also observe here (Table S3). The isomer shift and quadrupole of Fe(III) observed here are close to reported values of 0.32 mm/s^42,43^ and 0.7 mm/s,^42^ respectively. Note that there is no sign of magnetic hyperfine splitting in the Fe(III) component, in contrast to the 1.8 wt % sample in Bhide and Tambe,^42^ which suggests that the synthesis method utilized here did not lead to clustering of Fe(III) or formation of ferrite phases. For Fe(II), the isomer shift is in agreement with all earlier reports, and the quadrupole splitting for the minority Fe(II) phase is close to the splitting of 0.32–0.36 mm/s observed by Shirane et al.^40^ for Fe_x_Mg_1–x_O (0.10 < x < 0.75). An interesting contrast to the results by Shirane et al.^40^ is that they did not observe an Fe(II) component without quadrupole splitting, which is probably the result of different synthesis conditions.

### Effects of Relative Humidity over Short Periods

#### Secondary Phase Formation on (Mg,Fe)O in Solution

**Figure 1:**
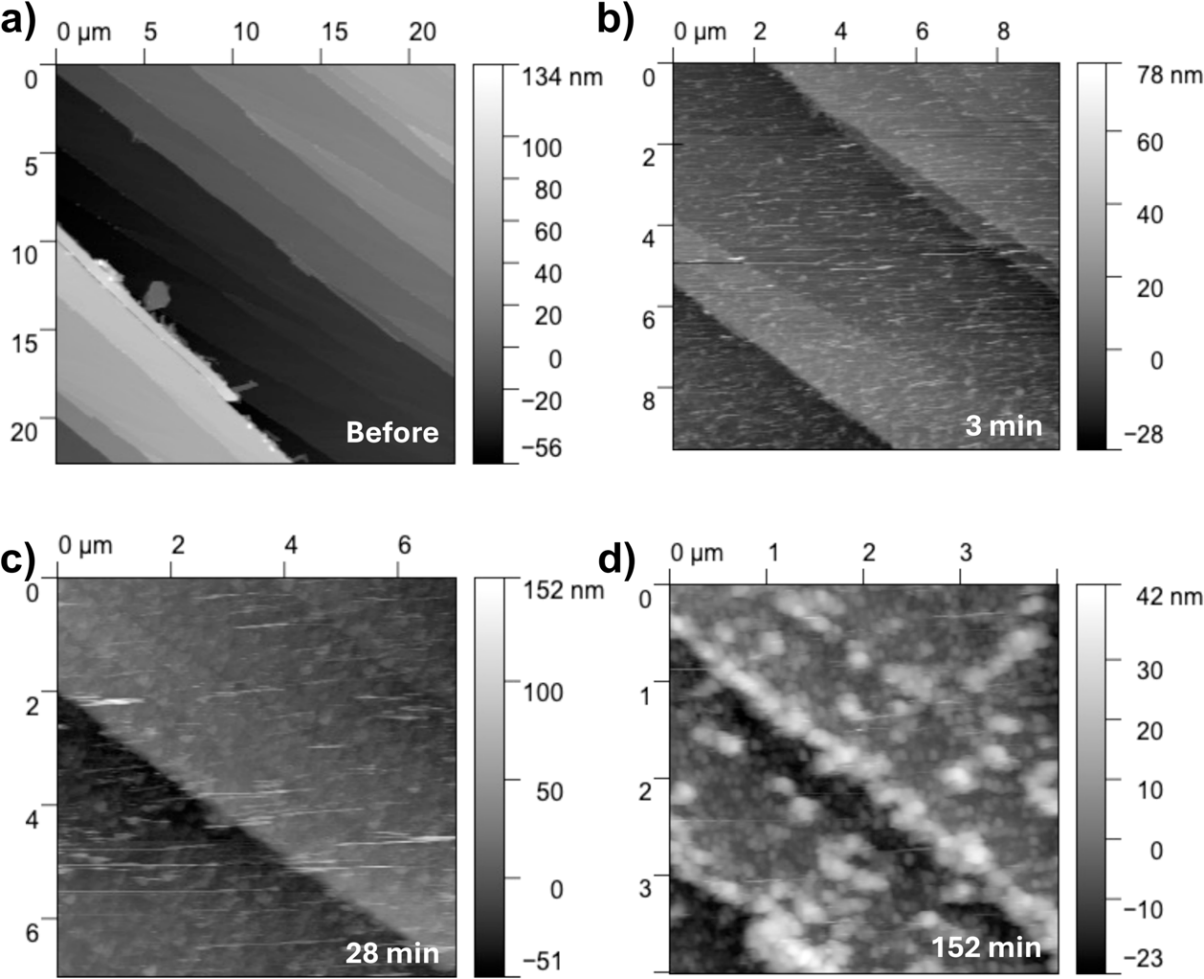
In situ AFM images of (Mg,Fe)O in contact with MgO-saturated solution, adjusted pH = 12.46. (a) Height mode AFM image of a freshly cleaved (Mg,Fe)O sample. (b) Small precipitates are visible after 3 min reaction with MgO-saturated solution. (c) After 28 min of reaction, the precipitates are larger. (d) After 152 min of reaction, large precipitates are visible.

To compare the reactivity of (Mg,Fe)O with that of pure MgO, AFM was used to characterize the formation of secondary phases in situ. Since previous studies showed rapid layer formation on pure MgO,^11,37,44^ an MgO-saturated solution with an adjusted pH of 12.46 was used to slow the reaction down and facilitate observations. Dry (Mg,Fe)O directly after cleaving has characteristic features associated with pure MgO.^37,45,46^ These are multilayer cleavage steps that are parallel to each other (Figure 1a). Within 3 min of exposure, a secondary phase forms, which is uniformly distributed on the surface of the (Mg,Fe)O (Figure 1b). After 28 min, the size of newly formed features on the surface increases to a mean value of 8.62 nm (Figure 1c). Feature size increases to an average size of ∼646 nm after 152 min of reaction time (Figure 1d).

#### Electron Microscopy Characterization of Thin Films Formed on (Mg,Fe)O

As the initial step in the formation of carbonates on MgO is the formation of a hydrated layer such as Mg(OH)_2_, we first explored the role of high relative humidity on (Mg,Fe)O samples by reacting cleaved samples in humid N_2_ (>95% RH) for 5–15 min for comparison with our previously published results on MgO.^11^ Using BF-TEM, we do not observe any noticeable reaction layer on (Mg,Fe)O (Figure S4), in contrast to our results on pure MgO, where a reaction layer is visible.^11^

**Figure 2:**
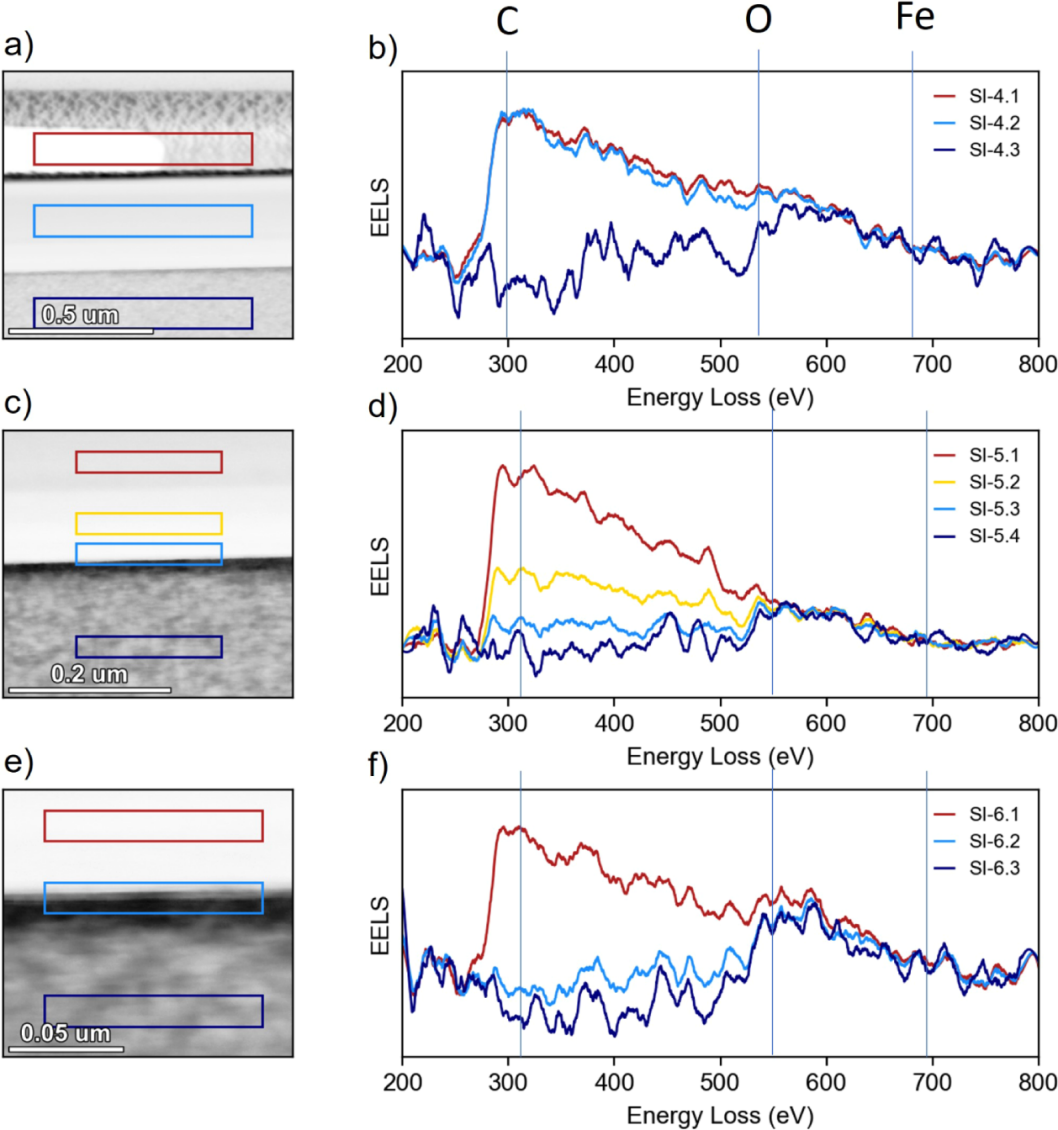
STEM-EELS maps of (Mg,Fe)O reacted overnight at 11% RH (sample name: (Mg,Fe)O 11p-2). (a), (c), and (e) show high angle annular dark field (HAADF) images of interface reaction layer. Spectraintegrated over the colored rectanglesare given by the graphs of respective colors in the figures (b), d), and (f). Red rectangles are carbon coating, yellow/light blue are interface, and dark blue are bulk (Mg,Fe)O).

**Figure 3:**
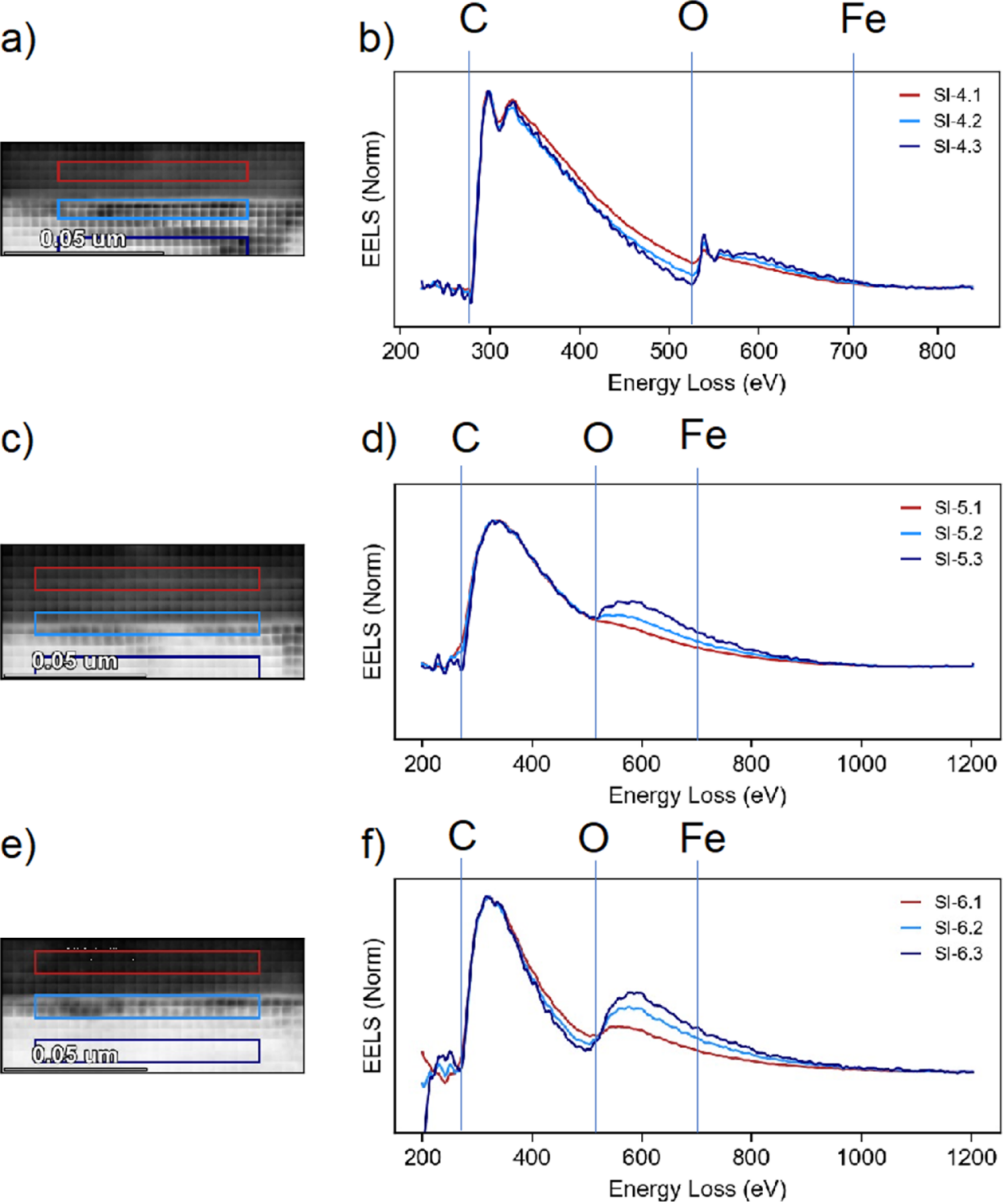
STEM-EELS maps of (Mg,Fe)O reacted at 11% RH for 4 h and a total of 15 min at >95% N_2_ (sample name: (Mg,Fe)O 11p-4h). (a), (c), and (e) show high angle annular dark field (HAADF) images of interface reaction layer. Spectra integrated over the colored rectangles are given by the graphs of respective colors in the figures(b), (d), and (f). Red rectangles are carbon coating, light blue are interface, and dark blue are bulk (Mg,Fe)O).

STEM-EELS spectrum images were acquired on the cross-section of the (Mg,Fe)O surface from the unreacted bulk to the C coating. Each of the colored rectangles on the images (Figures 2 and 3a,c,e) corresponds to an integrated EEL spectrum of the respective color (Figures 2 and 3b, d, f); areas of the rectangles are identical to facilitate more quantitative comparison of the elemental composition near the (Mg,Fe)O surface. Spectral range was selected to cover C K, O K, and Fe L edges with a dispersion level of 0.3 and 0.5 eV/channel. For both O K and Fe L, the signal was too weak to differentiate from the base noise level during acquisition. In the bulk solid (dark blue rectangle in Figures 2 and 3), no resolved C K peak at 284 eV is observed. In the case of Figure 2a, the spectrum in the bulk shows a higher signal intensity only due to the higher thickness of the sample in that area. A small amount of C is detected near the interface, with more C detected farther away from the interface, stemming from the C coating of the sample. No change in the region of O K edge at 532 eV or the Fe L3 edge at 710 eV was detected in the sample reacted overnight at 11–12% RH ((Mg,Fe)O 11p-2) (Figure 2). However, the sample reacted for 15 min in N_2_ at >95% RH ((Mg,Fe)O 11p-4h) shows a resolved O K edge: its signal can be seen to decrease from the bulk across the surface and into the C coating. The visible fine structure for C K edge (Figures 2a,b,c,d and 3a,b) is indicative of the constituent pi* and sigma* transitions being resolved, caused likely by smaller sample thickness in these areas compared to other panels; however, with or without the fine structure (such as 3c,d,e,f,), C K edge signal demonstrates the presence of C within the sampled area.

#### X-Ray Reflectivity Characterization of Thin Films Formed on (Mg,Fe)O

**Figure 4:**
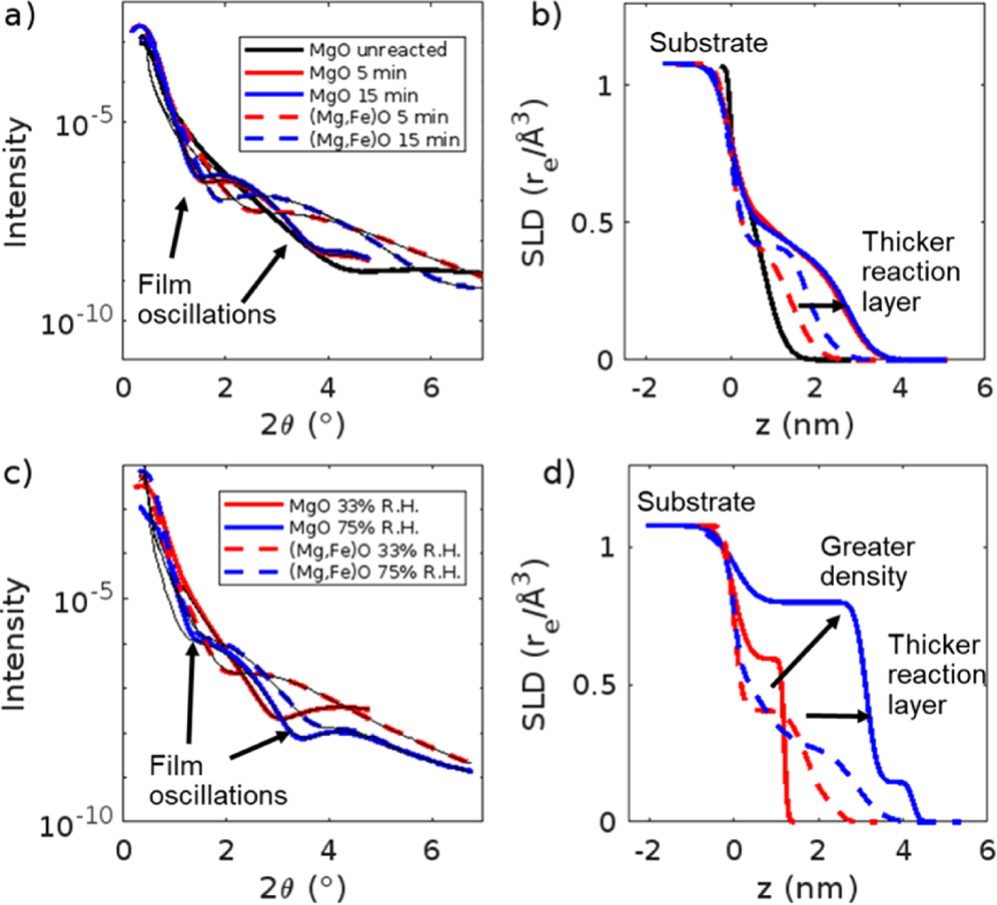
XRR profile (a) of MgO^11^ and (Mg,Fe)O samples reacted in humid N_2_ from 0 to 15 min and the scattering length density (SLD) profiles (b) from the fits of the data. XRR profile (c) for MgO^11^ and (Mg,Fe)O samples reacted in air at 33% RH and 75% RH for 8 days and their scattering length density (SLD) profiles (d) from the fits of the data. MgO data reproduced from reference.^11^ Copyright 2024 American Chemical Society.

While the TEM measurements provide two-dimensional information on reaction layer thickness across a small width (∼10–15 μm), XRR probes the reaction layer properties averaged over a lateral area of 500 μm × 100s–1000s of μm. In comparison with our previous results on MgO,^11^ the XRR curve for the (Mg,Fe)O sample has a minimum for the first oscillation at a larger 2θ value than MgO, even after 15 min of reaction (Figure 4a,b). This indicates the reaction layer for the (Mg,Fe)O forms more slowly than on pure MgO upon exposure to humid N_2._ The MgO samples required a two-layer model for fitting, with a denser layer near the MgO substrate followed by a less dense layer.^11^ The (Mg,Fe)O samples could be fit using a single-layer model (Table S4), which suggests that there is less variability within the sample as compared to the MgO sample. Based on the scattering length densities (SLD) derived from the fits (Figure 4b), the reaction layer thickens from ∼2.5 to ∼3 nm from 5 to 15 min on the (Mg,Fe)O sample, while the MgO sample has a reaction layer ∼4 nm thick after only 5 min, which does not increase from 5 to 15 min.^11^ This suggests that the MgO sample may be passivating over these short time periods, while the (Mg,Fe)O sample is not.

### Effects of Relative Humidity over Longer Time Periods

#### STEM and TEM Imaging

**Figure 5:**
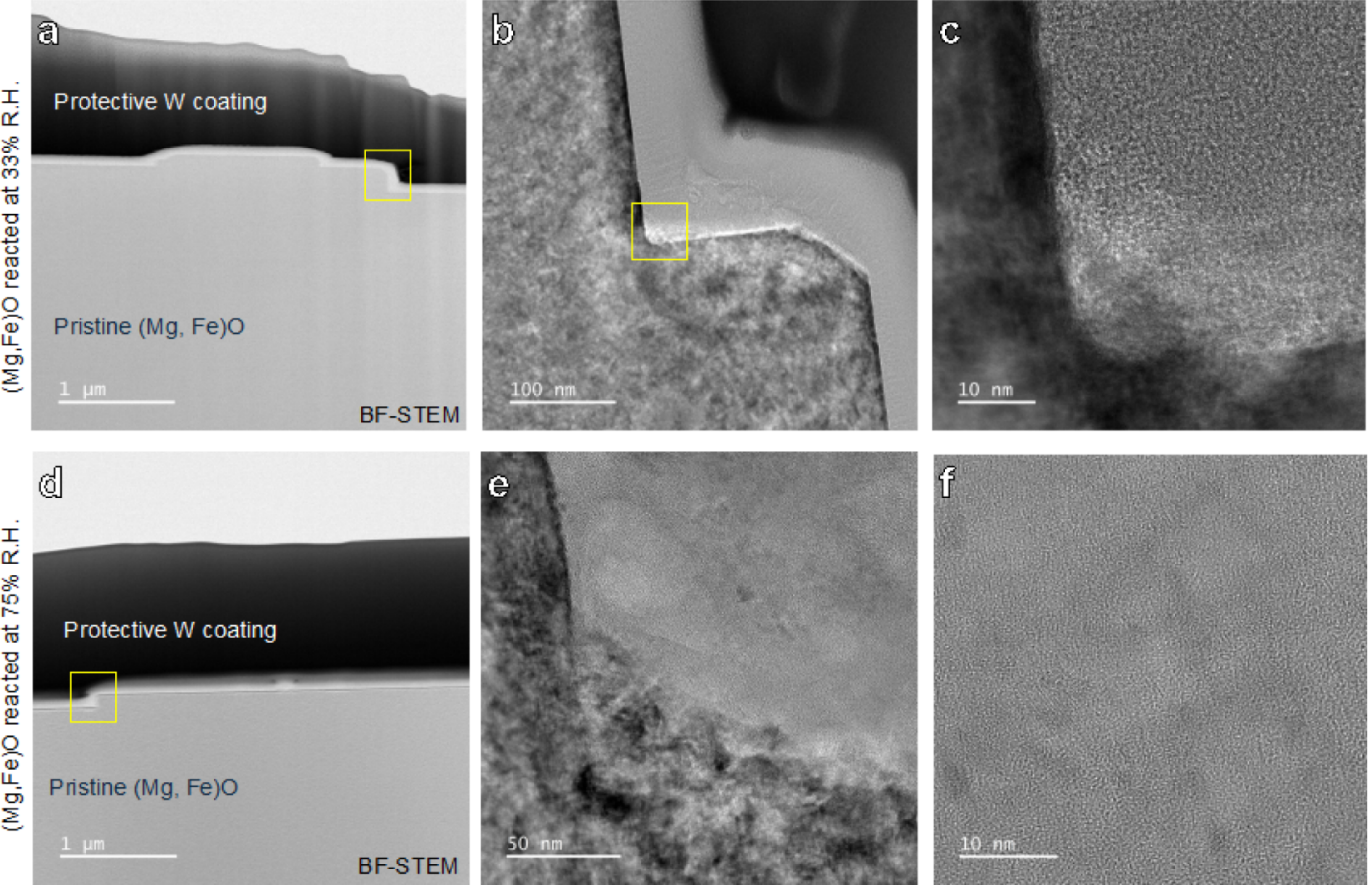
Electron microscopy characterization of (Mg,Fe)O reacted for 8 days in air at 33% RH (a–c) and at 75% RH (d–f).

Due to the slow initial reactivity of (Mg,Fe)O, longer-term ex situ experiments were set up to identify reaction layer formation. No secondary phases are evident on reacted (Mg,Fe)O samples using scanning electron microscopy (Figure S5a,b). In comparison to pure MgO samples,^11^ we do not observe a consistent reaction layer via TEM on (Mg,Fe)O in the presence of humidity in air when reacted for 8 days. Secondary phase formation is evident in TEM/STEM images only at steps (Figure 5), which are regions of higher defect density and therefore are more reactive. Locally, there is an ∼50 nm thick reaction layer (Figure 5c,f).

To identify the presence of crystalline phases in Figure 5 and f, a combination of non-negative matrix factorization (NMF)^47^ and sliding fast Fourier transform (FFT)^48^ analysis was used. First, we analyzed the crystalline part of the reaction layer for (Mg,Fe)O reacted at 33% RH (Figure 5c) using NMF. Based on NMF analysis, the reaction layer has a complex composition, and there are at least 8 components with distinct spatial distributions (these may include different orientations of the same phases). Details of the NMF analysis and component distribution map are given in Figure S6. The calculated FFT of Figure 5c shows split reflections at higher indices/low *d*-spacings, which correspond closely to what would be expected for the overlap or intergrowth of MgO (100) and magnesite (MgCO_3_) (221) (Figure S7). The radial distribution function of the calculated FFT of Figure 5c was compared with literature data for MgO, nesquehonite (MgCO_3_·3H_2_O), brucite (Mg(OH)_2_), magnesite (MgCO_3_), hydromagnesite (Mg_5_(CO_3_)_4_(OH)_2_·4H_2_O), artinite (Mg_2_(CO_3_)(OH)_2_·3H_2_O), and lansfordite (MgCO_3_·5H_2_O) to identify which other phases in addition to magnesite are present. While a previous study had observed the formation of dypingite,^3^ there is no structural data available to simulate the corresponding radial distribution function, so we were unable to evaluate our FFT data for the presence of this phase. Since the sample is highly oriented, not all peaks that are present in the powder diffraction patterns from the literature are observed in our measurements.

The HR-TEM image of (Mg,Fe)O reacted at 75% RH also shows visible lattice fringes, indicating crystalline phases. The NMF component analysis has 8 components. The sliding FFT analysis shows that the phases formed on (Mg,Fe)O reacted at 75% RH are overall less crystalline compared to the phases formed at 33% RH. This is evident from the fact that there are fewer higher-order reflections present and almost no variations in the range representing higher *d*-spacings. Comparing observed intervals with literature data indicates that it could be either hydromagnesite or nesquehonite; however, individual reflections are more consistent with hydromagnesite. A combination of NMF analysis results and sliding FFT indicates that part of the visible lattice fringes originates from MgO (components 4, 7, and 8).

To summarize, the (Mg,Fe)O reacted at 33% RH has a higher crystallinity of newly formed phases at step edges. Here, newly formed crystalline phases are directly located on top of the MgO. NMF and sliding FFT indicate that the newly formed phases are likely to be hydromagnesite, MgO, and magnesite. However, the (Mg,Fe)O reacted at 75% RH has poorer observed crystallinity, and the phases are likely MgO of different orientations and hydromagnesite but not magnesite.

#### X-Ray Reflectivity Characterization of Thin Films Formed on (Mg,Fe)O over Longer Time Periods

We previously determined that MgO reaction layer thickness and coverage increase with relative humidity and reaction time.^11,49^ Here, we performed similar experiments for the (Mg,Fe)O samples after 8 days of reaction in air at 33% and 75% RH. Based on the XRR profiles for the (Mg,Fe)O samples, the sample reacted in air for 8 days at 33% RH has an oscillation minimum at 2θ ∼ 2° (Figure 4c,d). However, the sample reacted in air for 8 days at 75% RH has two oscillation minima at 2θ ∼ 1.4° and 3.7°, which indicates the reaction layer is thicker in 75% RH than in 33% RH. In contrast to our samples reacted for 15 min in humid N_2_ (Figure 4a, b), both the MgO^11^ and (Mg,Fe)O samples required a two-layer model for fitting, with a denser layer near the MgO substrate followed by a less dense layer (Table S5).

The SLD profiles (Figure 4d) show both thickening of the reaction layers and changes in densities. When comparing our (Mg,Fe)O samples to previous MgO samples reacted for 8 days,^11^ both substrates have a greater reaction layer thickness at 75% RH compared to 33% RH, but at 75% RH, the MgO sample has a thicker reaction layer as compared to (Mg,Fe)O (Figure 4c,d). Based on the SLD plots, the reaction layer thickness for the (Mg,Fe)O 75% RH is ∼4 nm compared with ∼3 nm for the 33% RH sample. The reaction layer thickness based on the SLD profile for the (Mg,Fe)O at 33% RH is twice as large as compared with the MgO sample (∼3 vs 1.5 nm).^11^ However, the reaction layer thickness for the MgO sample at 75% RH is 17.5% greater than that on the (Mg,Fe)O sample (∼4.7 vs 4 nm), demonstrating the importance of humidity on reactivity.

#### GIXRD Analysis of the Impact of Humidity on Secondary Phase Formation on (Mg,Fe)O

GIXRD was utilized to detect if crystalline phases were present in the film. Similar to our previous results,^11^ the majority of the spectra have five peaks corresponding to the powder XRD pattern of periclase (MgO) (Figure S8). The periclase signals are caused by either X-ray penetration in the upper section of the MgO or particles on the surface. Given that the samples were cleaved during preparation, a small quantity of MgO powder on the surface is not surprising. In many of our samples, we observed both sharp peaks, indicative of crystalline material, and broad peaks, which indicate amorphous or nanocrystalline material.

Previously, we observed a broad peak around 2θ = 50° for our MgO samples reacted for 8 days in 75% and 33% RH.^11^ Our (Mg,Fe)O samples were reacted at the same time and in the same reactor vessels; however, in general, these samples do not exhibit broad peaks, aside from possibly the 75% RH sample. For the 33% RH sample, there is a shoulder for the second and third periclase peaks, and there appears to be a signal at 2θ > 60°, which is beyond our measurements. For the 75% RH sample, there are no secondary peaks, but the signal is somewhat broader around the second peak, potentially indicating an amorphous or nanocrystalline material. However, it is less indicative of an amorphous broad feature than that of pure MgO. Combined with the XRR data, this suggests that the (Mg,Fe)O samples may be less reactive.

### Effects of CO_2_

#### STEM

Ex situ experiments on the effect of Fe-doping on MgO carbonation were conducted for a duration of 30 days in CO_2_ at 75% RH and compared to MgO samples carbonated under the same conditions.^49^ On the surface of the (Mg, Fe)O, newly formed nuclei are visible using scanning electron microscopy. They preferentially form along steps but also distribute on terraces (Figure S5c). Image analysis of SEM images showed that they had an average area of ∼300 nm^2^ for pure MgO^49^ and ∼100 nm^2^^2^ on (Mg,Fe)O.

**Figure 6:**
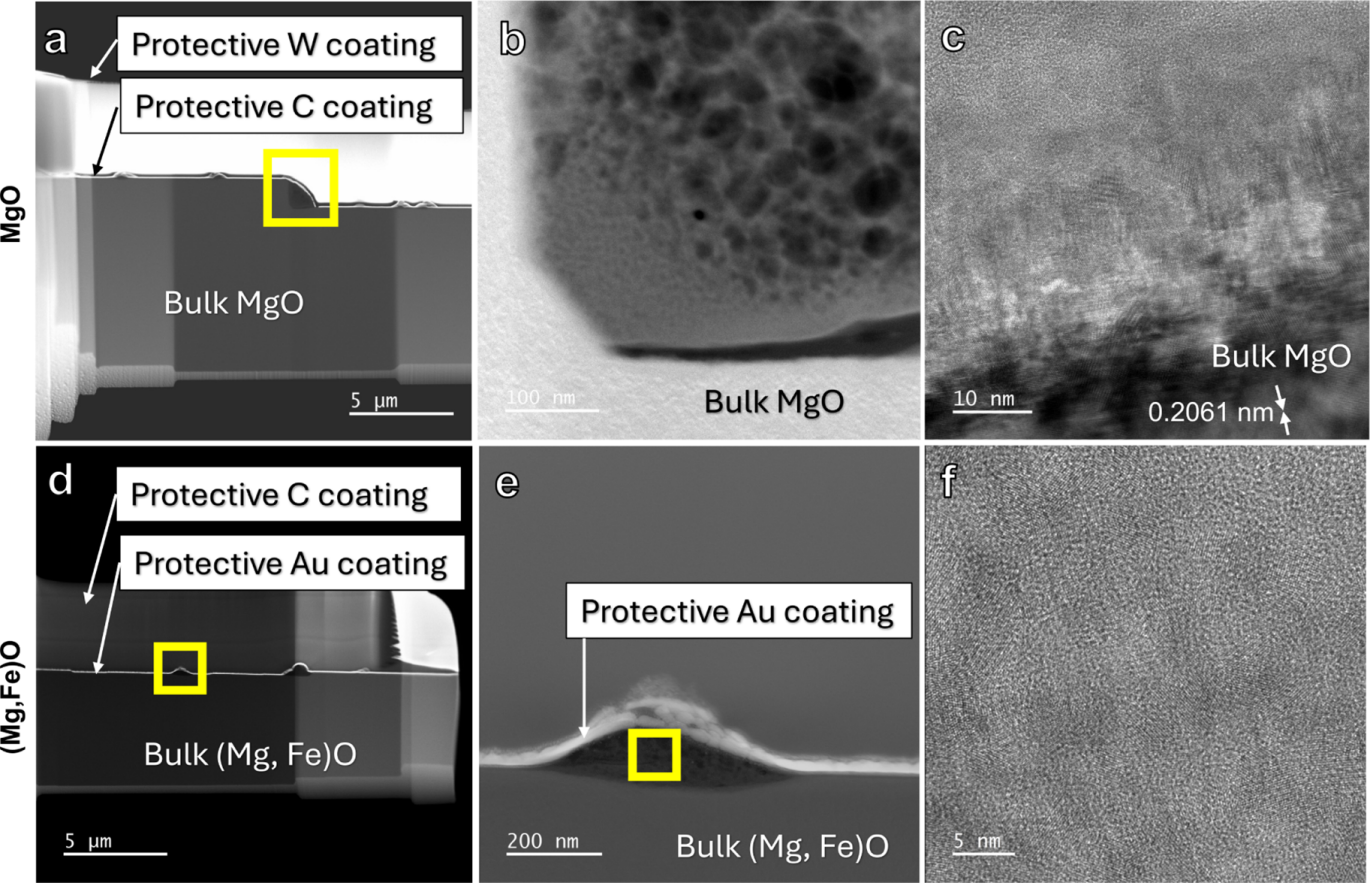
Electron microscopy results of MgO (a–c) and (Mg,Fe)O (d–f) ex situ carbonation experiments. Samples were reacted at 75% RH for 30 days in the presence of CO_2_. Image (a)–(c) are from Yang et al., 2025.^49^ MgO data reproduced from reference.^49^ Copyright 2025 American Chemical Society.

TEM samples were prepared from several nuclei using the FIB lift-out method, leading to a cross-section of nuclei. HAADF-STEM imaging showed that the newly formed phase is highly porous (Figure 6). Furthermore, it was easily damaged by the electron beam in STEM mode, indicating that the material is different from MgO, which is generally stable under the electron beam. Possibly, this could be due to the formation of hydrated phases. BF-TEM imaging shows that the newly formed phases contain nanocrystallites. Using NFINDR analysis of FFT from HR-TEM images in Figure 6c, the phase identified is likely to be barringtonite (MgCO_3_·2H_2_O).^49,50^

#### X-Ray Reflectivity Characterization of Thin Films Formed on (Mg,Fe)O in the Presence of CO_2_

**Figure 7:**
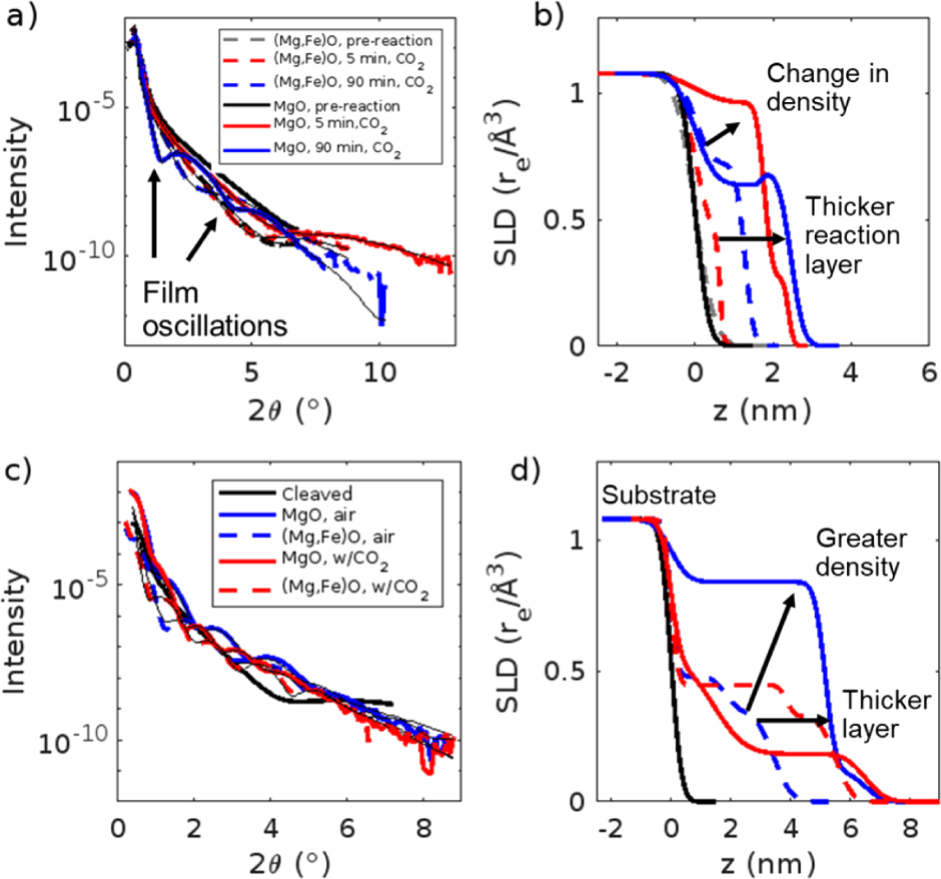
XRR profile (a) of MgO^49^ and (Mg,Fe)O samples reacted in humid CO_2_ for 0–90 min and their scattering length density (SLD) profiles (b) from the fits of the data. XRR profile (c) of MgO^49^ and (Mg,Fe)O samples reacted under 75% RH in air or CO_2_ and their scattering length density (SLD) profiles (d) from the fits of the data. MgO data reproduced from reference.^49^ Copyright 2025 American Chemical Society.

We conducted additional in situ and ex situ experiments to determine if iron will disrupt phase formation in the presence of CO_2_ and compared these results with our previous measurements on MgO.^49^ For our in situ experiments where the sample was exposed to humid CO_2_, the XRR profile for the (Mg,Fe)O sample initially has an oscillation with a minimum at 2θ ∼ 4.5° after 5 min (Figure 7a). The oscillation shifts to a lower 2θ value as the reaction time progresses (Figure S9), indicating the thickness of the reaction layer increases; after 90 min, the minimum is at 2θ ∼ 2.7° (Figure 7a,b). After 90 min of reaction, the sample was reacted with deionized water, followed by 30 min of humid CO_2_ to determine if the reaction layer could be removed and regrown. However, there is not a significant difference in the location of the oscillation (Figure S10), suggesting limited removal of the film. This differs from our previous measurements on MgO, in which the reaction layer was removed after 2 min of exposure to deionized water.^49^

XRR profiles measured on our (Mg,Fe)O sample have shallower, less defined oscillations that are located at higher 2θ values as compared to our previous MgO results.^49^ The (Mg,Fe)O data could be fit using a single-layer model (Table S6), but the MgO samples required a two-layer model for fitting, with a denser layer near the MgO substrate followed by a less dense layer.^49^ This suggests that the presence of iron decreases the sample reactivity. Based on model fitting, there is both thickening of the reaction layers and changes in density over time. From 5 to 90 min, the reaction layer on (Mg,Fe)O thickens from ∼0.8 to ∼1.8 nm, and the reaction layer on MgO thickens from ∼2 nm to ∼3 nm^49^ (Figure 7a,b). This suggests the initial reaction on (Mg,Fe)O is slower than that of MgO. The reaction layer on (Mg,Fe)O is also less dense than that of MgO.^49^

To understand longer-term behavior, we measured (Mg,Fe)O samples reacted in CO_2_ at 33% and 75% RH or in air at 75% RH for 30 days. In comparison to the (Mg,Fe)O sample reacted in air for 30 days, the XRR profile for the (Mg,Fe)O sample reacted in CO_2_ for 30 days has an oscillation minimum at a lower 2θ value, as well as oscillations closer together (Figure 7c). Model fits were performed (Table S7) to indicate the reaction layers on (Mg,Fe)O and MgO^49^ reacted in CO_2_ are ∼6–8 nm thick, though the reaction layer for the (Mg,Fe)O sample is ∼0.5 nm thinner than that of MgO reacted under the same conditions (Figure 7d). The reaction layer on the (Mg,Fe)O sample in air is also ∼2 nm thinner than the reaction layer on the (Mg,Fe)O sample reacted with CO_2_ (Table S7). In contrast, our previous results on MgO^49^ did not have significant differences in the reaction layer thickness in air as compared with CO_2_ at 75% RH, though the density of the reaction layer was greater when formed in air than CO_2_. The reaction layers formed on (Mg,Fe)O in CO_2_ at 75% RH are less dense than our previous results on MgO^49^ (Table S7), consistent with our measurements in air and humid N_2_. The (Mg,Fe)O sample reacted in CO_2_ at 33% RH has no apparent oscillations, suggesting there may not be a reaction layer present (Figure S11). In contrast, MgO reacted in CO_2_ for 30 days at 33% RH has a reaction layer thickness of ∼6 nm, approximately 2 nm thicker than the reaction layer for MgO reacted in air under similar conditions.^49^ This suggests that iron is inhibiting reaction layer formation.

#### GIXRD Analysis of (Mg,Fe)O in the Presence of CO_2_

Similar to our samples reacted in air, the majority of our GIXRD measurements exhibited peaks characteristic of periclase (Figures S12 and S13). The (Mg,Fe)O sample reacted in humid CO_2_ (Figure S12b) had fewer of the periclase peaks than the MgO sample^6,49^ (missing the peaks at 2θ = ∼29° and ∼60°), though this may have occurred if there was less powder that formed during cleaving. After reacting the sample with deionized water, the peaks at 2θ ∼ 58° and 60° disappeared for the MgO^49^ and (Mg,Fe)O samples, possibly due to particle removal postexposure to water. Overall, for the samples reacted in situ, there was not a significant change in the number and location of peaks present, suggesting that there might not be significant formation of crystalline secondary phases.

In contrast, the samples reacted for 30 days exhibited more differences in the GIXRD (Figure S13). For example, both the MgO (Figure S13a)^49^ and the (Mg,Fe)O (Figure S13c) samples reacted in 33% RH in CO_2_ for 30 days exhibited many peaks in addition to the periclase peaks, possibly due to the precipitation of an amorphous phase. For both samples, after rinsing with deionized water, the additional peaks were no longer present, suggesting the removal of the phase. However, the (Mg,Fe)O sample retained a broad peak around the second periclase peak even after undergoing a rinsing process with water, possibly due to the presence of amorphous material (Figure S13c). For the 30-day sample reacted in air, 33% RH the (Mg,Fe)O sample exhibited a broad peak between 2θ = 25° and 35°, again possibly due to the presence of amorphous material.

#### Mechanism of Inhibition

Overall, our results demonstrate that iron incorporated into MgO will inhibit carbonation reactions on MgO(100), although the mechanisms of reaction layer formation are similar on samples with and without iron present. In general, the presence of iron reduces how quickly the reaction layer grows and thus the amount of growth of the reaction layer in a given period of time, as well as the density of the reaction layer that forms. Previously, we demonstrated that passivation can occur quickly on MgO;^4,11,49^ however, here the iron appears to inhibit passivation. That is, for MgO samples reacted in situ in either N_2_^11^ or CO_2_,^49^ passivation occurs after minutes, while the (Mg,Fe)O samples continue growing under similar time periods. Since the reaction layer formation mechanisms appear similar for MgO and (Mg,Fe)O, it could be that (Mg,Fe)O still passivates but requires longer time periods than those we have studied here.

Our TEM data show that secondary phases on (Mg,Fe)O are concentrated along defect-rich regions like steps. In the presence of CO_2_, the reaction layer was porous and had nanocrystallites present within these newly formed phases. Our findings show that iron impurities limit carbonation and film formation on MgO surfaces, which is consistent with previous studies on brucite,^31^ where it was found that increasing iron(II) substitution reduced carbonation efficiency in both oxic and anoxic environments. As a result, our findings, together with earlier studies, provide evidence of a reduction in the initial carbonation rate of the MgO samples containing iron impurities, which may inhibit the CO_2_ uptake. However, if passivation takes longer to occur, it leads to the intriguing possibility that there may still be a net increase in the level of CO_2_ uptake at long time scales despite the reduced initial rate.

Computational simulations demonstrated that an OH^–^ layer is present on MgO and (Mg,Fe)O surfaces, at which adsorption of (bi)carbonate is favorable.^27^ While similar mechanisms of adsorption occur on MgO and (Mg,Fe)O, the H-bond lifetimes are shorter on (Mg,Fe)O than on MgO, possibly due to the preferential leaching of Fe, which introduces acidity and reduces the amount of OH^–^ available at the surface.^27^ In contrast, when millimolar concentrations of dissolved iron are present in solution during MgO hydroxylation, nanophase iron oxides form that enhance the carbonation of MgO.^33^ If leaching out of iron from the samples used in our study occurs, it is likely below the threshold for significant formation of iron oxides, as they were neither observed in our TEM nor GIXRD measurements. Therefore, we hypothesize that the iron present may be leaching out and contributing to changes in pH at the surface that, in turn, disrupts the adsorption of hydroxide and (bi)carbonate.

#### Implications for Direct Air Capture of CO_2_

Our study shows that the incorporation of iron impurities into the MgO samples disrupts the hydrated phase formation and inhibits film growth during both hydroxylation and carbonation of the surface at ambient temperatures. This implies that naturally occurring impurities, such as iron, present in MgO play a significant role as a limiting factor for DAC conditions. For example, when contrasting the reaction thickness between our 90-min in situ samples reacted at >95% relative humidity and our 30-day ex situ samples reacted at 75% relative humidity, we note a greater thickness on the MgO samples compared to the (Mg,Fe)O samples, despite both being subjected to identical conditions and reaction durations. Given that impurities naturally exist in MgO deposits crucial for DAC mineral looping techniques, the purity of the MgO must be taken into consideration when employing DAC. A high percentage of iron impurities in MgO might diminish the CO_2_ removal efficiency and extend the time required for looping cycles.

There appear to be opposite effects on the carbonation of MgO if the iron is incorporated compared with iron being present in the solution phase during hydration of MgO prior to carbonation. In this study, we found that iron incorporated into the MgO as (Mg,Fe)O inhibits hydration and carbonation. In another recent study, we investigated the effect of dissolved iron present during the hydration of MgO prior to subsequent carbonation (Weber et al., 2025).^33^ In those results, the presence of dissolved iron leads to an increase in carbonationdue to the formation of a nanoscale iron oxide phase as indicated by Mössbauer and magnetometry measurements. These findings indicate that it will be crucial to identify impurities present in both solution and solid phases and their effects for accurate life cycle analysis of mineral looping processes.

## Conclusions

Our results demonstrate that while (Mg,Fe)O has similar reaction mechanisms as pure MgO in humid N_2_ and humid CO_2_, the presence of iron inhibits the reaction rate over both short (minutes) and long (days to a month) time scales. Relative humidity increases the rate of reaction of (Mg,Fe)O, similar to previous observations on MgO,^11^ leading to thicker reaction layers of greater density. However, surface passivation was not observed for the (Mg,Fe)O samples, likely due to the slower reaction rate. Electron microscopy results showed the formation of hydromagnesite after 8 days of reaction in ambient air at 33% and 75% RH with products at 33% RH being more crystalline. Additionally, magnesite as a product was only observed at 33% RH. Overall, our findings show that the iron impurity in the MgO disrupts hydrated phase formation and inhibits film growth during both hydroxylation and carbonation of the surface. This suggests that naturally occurring impurities in MgO potentially reduce CO_2_ uptake during direct air capture and require longer reaction times to capture comparable quantities of CO_2_.

## Data Availability

Data associated with the manuscript is available at doi: 10.17632/d53h3r7dng.1

## Supporting Information Available

Tables of XRR reaction conditions and fitting, electron microprobe data, Mössbauer spectral parameters; figures of BSE and SEM images, diffractograms, NMF component analysis, and XRR and GIXRD patterns (PDF)
