# Supplementary material for: Inhibition of Reaction Layer Formation on MgO(100) by Doping with Trace Amounts of Iron
Source: J Phys Chem C Nanomater Interfaces. 2025 Feb 12;129(7):3457–68. doi: 10.1021/acs.jpcc.4c06311 (PMC11848909; doi:10.1021/acs.jpcc.4c06311)
Supplement: Supplementary file 1 — jp4c06311_si_001.pdf [file jp4c06311_si_001.pdf]

Supporting Information For Inhibition of Reaction Layer Formation on MgO(100) by Doping with Trace Amounts of Iron

Gabriela Camacho Meneses[1], Juliane Weber[2], Raphaël P. Hermann[3], Anna Wanhala[4], Joanne E. Stubbs[4], Peter J. Eng[4,5], Ke Yuan[2], Albina Y. Borisevich[6], Matthew G. Boebinger[6], Tingting Liu[2], Andrew G. Stack[2], Jacquelyn N. Bracco[1,7*]

[1]School of Earth and Environmental Sciences, Queens College, City University of New York, Queens, NY, 11367-0904, USA

[2]Chemical Sciences Division, Oak Ridge National Laboratory, Oak Ridge, TN, 37831, USA

[3]Materials Science and Technology Division, Oak Ridge National Laboratory, Oak Ridge, TN, 37831, USA

[4]Center for Advanced Radiation Sources, The University of Chicago, Chicago, IL, 60637, USA

[5]James Franck Institute, The University of Chicago, Chicago, IL, 60637, USA

[6]Center for Nanophase Materials Sciences, Oak Ridge National Laboratory, Oak Ridge, TN, 37831, USA

[7]Earth and Environmental Sciences, Graduate Center, City University of New York, New York, NY, 10016-4309, USA

*Corresponding author email: jbracco@qc.cuny.edu

1. Methods

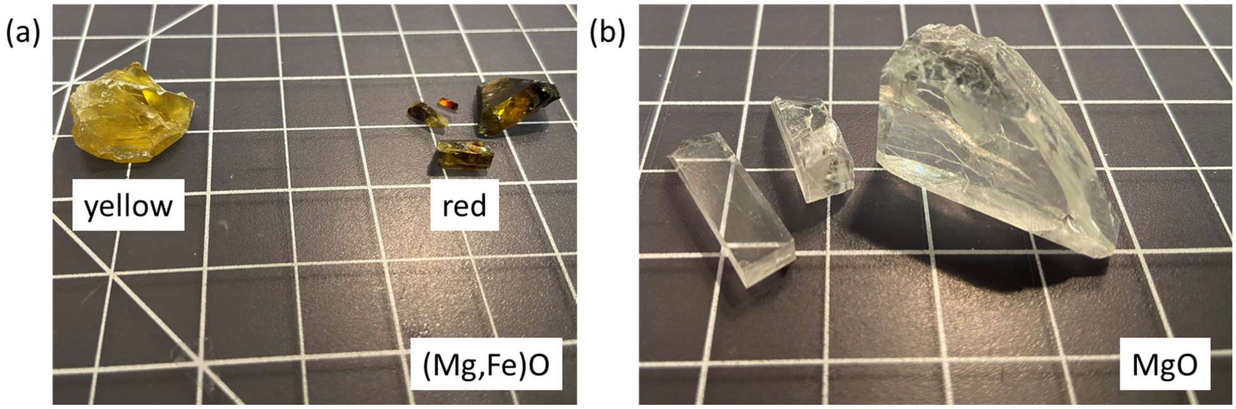

Fig. S1. Images of synthesized (a) (Mg,Fe)O yellow and red samples and (b) MgO samples for comparison. The squares on the grid are 0.5 inch by 0.5 inch.

Table S1. Summary of reaction conditions, times, and 2θ angle for the X-ray reflectivity (XRR) measurements. MgO data reproduced from reference [1]. Copyright 2025 American Chemical Society.

**Sample Type**	**Sample Name**	**Reaction Condition**	**Reaction Times**
(Mg,Fe)O	MgOFe_8d_33pRH	Air (R.H. = 33%)	8 Days
(Mg,Fe)O	MgOFe_8d_75pRH	Air (R.H. = 75%)	8 Days
(Mg,Fe)O	MgOFe_8d_75pRH_B	Air (R.H. = 75%)	8 Days
(Mg,Fe)O	MgOFe_100_11p_4h	Humid N_2 (R.H. > 95%)	5, 10, 15 Minutes
MgO	MgO_10p_dry	Humid CO_2 (R.H. > 95%)	5, 10, 15, 20, 30, 60, 90 Minutes
MgO	MgO_10p_dry	DI water	2 Minutes
MgO	MgO_10p_dry	Humid CO_2 (R.H. > 95%)	30 Minutes
MgO	MgO_10p_dry	DI water	2 Minutes, 2 Minutes
MgO	MgO_10p_dry	Humid CO_2 (R.H. > 95%)	30 Minutes
(Mg,Fe)O	MgOFe_8p_dry	Humid CO_2 (R.H. > 95%)	5, 10, 15, 20, 30, 60, 90 Minutes
(Mg,Fe)O	MgOFe_8p_dry	DI water	2 Minutes
(Mg,Fe)O	MgOFe_8p_dry	Humid CO_2 (R.H. > 95%)	30 Minutes
(Mg,Fe)O	MgFeO33_1month_CO2_s2	CO_2 (R.H. =33%)	30 Days
(Mg,Fe)O	MgFeO75_1month_CO2_s2	CO_2 (R.H. =75%)	30 Days
(Mg,Fe)O	MgFeO75_1month_s2	Air (R.H. =75%)	30 Days
MgO	MgO33_redo_1month_CO2_s1	CO_2 (R.H. =33%)	30 Days
MgO	MgO33_1month_s2	Air (R.H. =33%)	30 Days
MgO	MgO75_1month_CO2_s3	CO_2 (R.H. =75%)	30 Days
MgO	MgO75_1month_s1	Air (R.H. =75%)	30 Days

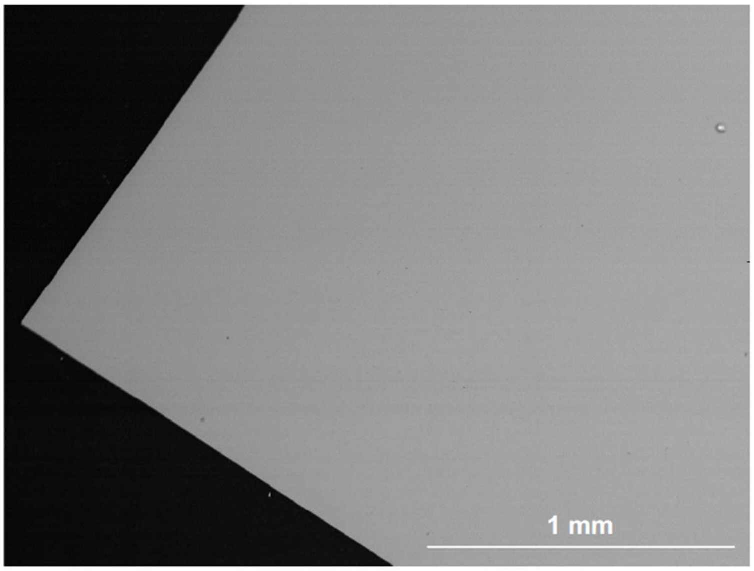

Fig. S2. BSE image of a yellow (Mg,Fe)O sample that shows no contrast variations, indicating the iron is homogenously distributed.

2. Results: Materials Characterization

Table S2. Electron microprobe analysis of a representative number of points. BSE image of sample is provided in Fig. S2. Bdl = below detection limit

	Weight%					Oxide				Detection limit [ppm]		
Point	Mg	Ca	Fe	O	Total	MgO	CaO	FeO	Total	Mg	Ca	Fe
1	60.0294	0.0096	0.556	39.6804	100.2754	99.5466	0.0134	0.7153	100.2754	365	165	220
2	59.9774	0.0071	0.5459	39.6423	100.1727	99.4604	0.01	0.7023	100.1727	359	165	214
3	60.0788	0.0003	0.5417	39.705	100.3257	99.6285	0.0004	0.6969	100.3257	385	168	212
4	60.1497	bdl	0.5236	39.7464	100.4197	99.7461	bdl	0.6736	100.4197	359	bdl	215
5	60.2685	bdl	0.5368	39.8284	100.6337	99.9432	bdl	0.6905	100.6337	353	bdl	207
6	60.0278	0.0008	0.5176	39.6648	100.2109	99.5439	0.0011	0.6659	100.2109	379	172	216
7	59.9488	0.0091	0.513	39.6148	100.0856	99.4129	0.0127	0.6599	100.0856	360	162	219
8	59.9171	0.0087	0.492	39.5878	100.0056	99.3605	0.0122	0.6329	100.0056	340	157	211
9	59.9173	0.0033	0.509	39.5906	100.0201	99.3607	0.0046	0.6549	100.0201	351	169	211
10	59.8342	0.0099	0.5535	39.5513	99.9489	99.223	0.0138	0.712	99.9489	349	164	206
11	59.8806	bdl	0.555	39.5783	100.0138	99.2998	bdl	0.714	100.0138	362	bdl	215
12	59.865	0.0057	0.5663	39.5736	100.0107	99.2741	0.008	0.7286	100.0107	355	166	209
13	59.9014	0.0064	0.5413	39.5907	100.0398	99.3345	0.0089	0.6964	100.0398	358	162	204
14	59.9398	0.0043	0.5151	39.6076	100.0667	99.3981	0.006	0.6627	100.0667	374	169	215
15	59.9612	0.0042	0.4942	39.6156	100.0752	99.4335	0.0058	0.6358	100.0752	367	166	213
16	59.979	0.0069	0.4883	39.6267	100.1009	99.4631	0.0097	0.6282	100.1009	367	165	216
17	59.9057	0.0014	0.4229	39.5575	99.8876	99.3415	0.002	0.5441	99.8876	395	169	223
18	59.8673	0.019	0.4613	39.5503	99.8979	99.2778	0.0266	0.5935	99.8979	359	158	217
19	59.7201	0.0039	0.5271	39.4662	99.7172	99.0337	0.0055	0.6781	99.7172	360	163	209
20	59.8372	0.0108	0.5223	39.5447	99.915	99.2279	0.0151	0.6719	99.915	354	162	217
21	59.665	bdl	0.5082	39.423	99.5962	98.9424	bdl	0.6538	99.5961	392	bdl	214
22	59.7246	bdl	0.5246	39.4669	99.7162	99.0412	bdl	0.675	99.7162	358	bdl	210
23	59.5945	0.0061	0.4954	39.3753	99.4713	98.8255	0.0086	0.6373	99.4713	355	164	215
24	59.8478	0.0055	0.4838	39.5385	99.8755	99.2454	0.0077	0.6224	99.8755	362	165	218
25	59.78	bdl	0.4481	39.4815	99.7096	99.1331	bdl	0.5765	99.7095	347	bdl	208
26	59.9738	0.0092	0.4185	39.6042	100.0057	99.4544	0.0129	0.5384	100.0057	366	161	216
27	59.7581	0.006	0.4378	39.4664	99.6683	99.0967	0.0084	0.5632	99.6683	348	165	216
28	59.8791	bdl	0.4557	39.5489	99.8836	99.2974	bdl	0.5862	99.8836	344	bdl	207
29	59.9962	0.0044	0.4328	39.6211	100.0546	99.4915	0.0062	0.5568	100.0546	341	169	217

Table S3 Mössbauer spectral parameters from fits to the data in Fig. S3. The total area is in %-effect.mm/s, the % indicate relative spectral areas of the species, and the isomer shift, δ, full-width at half-maximum, Γ, and quadrupole splitting, ΔE_Q, are in mm/s. For Fe(II) the isomer shift is constrained to be equal for two components and the quadrupole splitting is zero for the first Fe(II) component.

	Area	Fe(III)				Fe(II)					
Sample		%	δ	Γ	ΔE_Q	%	δ	Γ	%	Γ	ΔE_Q
'Yellow'	6.2(2)	24(2)	0.32(2)	0.48(6)	0.60(3)	71(5)	1.049(2)	0.27(1)	5(3)	0.25(1)	0.45(8)
'Red'	10.4(1)	8(1)	0.39(1)	0.50(2)	0.79(2)	65(1)	1.053(1)	0.34(1)	27(1)	0.27(1)	0.41(1)

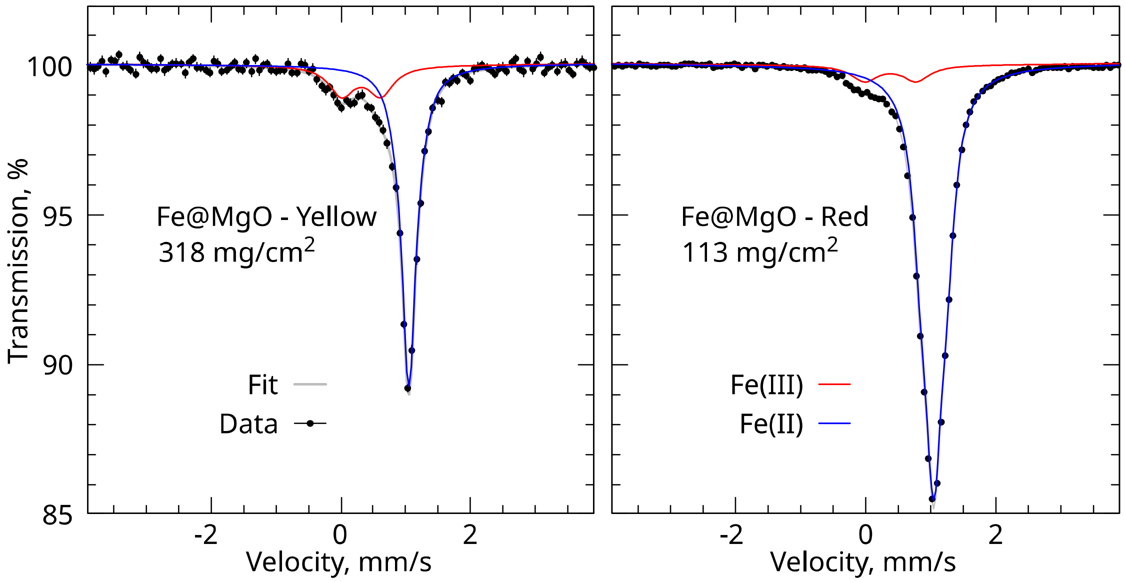

Fig. S3. Room-temperature Mössbauer spectra of Fe:MgO samples and their fits. The Fe(III) – red – component is a doublet, whereas the Fe(II) component is a sum of a doublet and a singlet. See Table S3.

3. Results: Effects of Humidity Over Short Time Periods

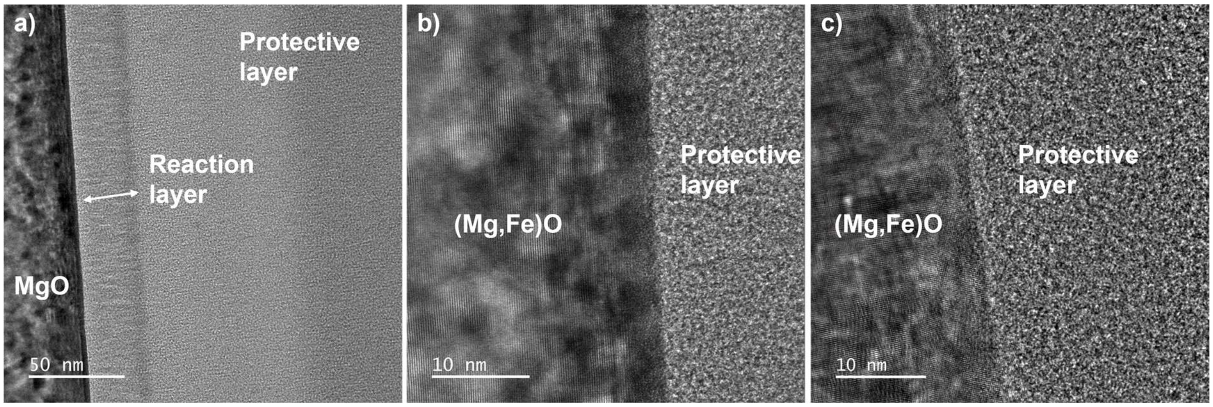

Fig. S4. BF-TEM images of reaction layer on (a) pure MgO (100), (b) (Mg,Fe)O reacted overnight at 11% R.H (sample name: (Mg,Fe)O 11p-2), and (c) of (Mg,Fe)O reacted at 11% R.H. for four hours and a total of 15 minutes at >95% N2 (sample name: (Mg,Fe)O 11p-4h). Figure (a) reproduced from reference [2], Copyright 2024 American Chemical Society.

Table S4. XRR fit results, film density (ρ), thickness (d), roughness (σ) and goodness of fit (χ2), for (Mg,Fe)O sample reacted in humid N_2 for 0-15 minutes.

R.H., time	layer 2			layer 1			Substrate	χ2
	ρ (g/cm^3) (±)	d (Å) (±)	σ (Å) (±)	ρ (g/cm^3) (±)	d (Å) (±)	σ (Å) (±)	σ (Å) (±)	
10%				2.366<br>0.002	7.34<br>0.01	4.28<br>0.01	0.50	15.99
>95%, 5 min CO_2, 10 Min Dry N_2				1.4<br>0.4	14.14<br>0.03	4.90<br>0.02	2.190<br>0.005	12.18
>95%, 10 min CO_2, 10 Min Dry N_2	1.72<br>0.01	5.37<br>0.05	5.83<br>0.05	1.5<br>0.7	12.28<br>0.03	0.80<br>(Fixed)	2.42<br>0.01	11.47
>95%, 15 min CO_2, 10 Min Dry N_2	0.89<br>0.02	3.4<br>0.1	4.75<br>0.05	1.3<br>0.5	17.22<br>0.03	2.17<br>0.08	2.579<br>0.005	9.51

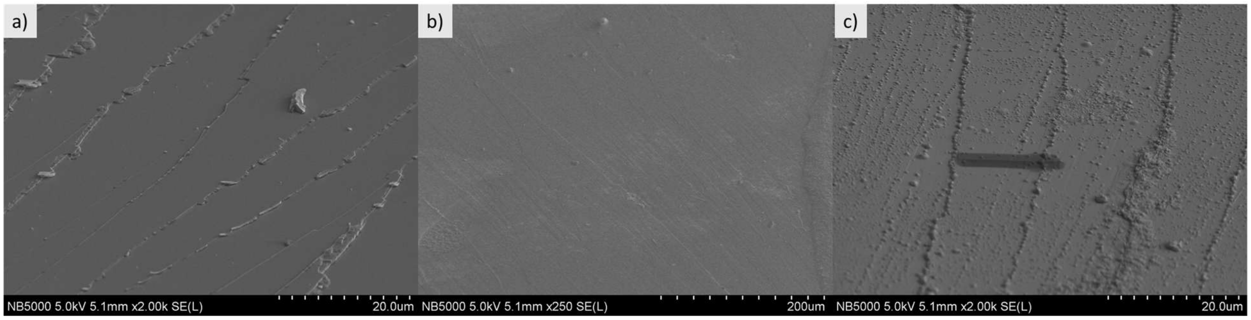

Fig. S5. SEM images after 8 days of reaction in air at a) 33% and b) 75% R.H. showing no evidence of a reaction layer on (Mg,Fe)O. (c) SEM image of (Mg,Fe)O after 30 days of reaction in CO_2 after 30 days at 75% R.H. showing formation of particles on the surface.

NMF analysis details

NMF analysis was performed on diffractograms of 256x256 image patches; the center portion of each diffractogram of 128x128 was used for analysis. Step size for the patch window on the original 4096x4096 image was 8 pixels.

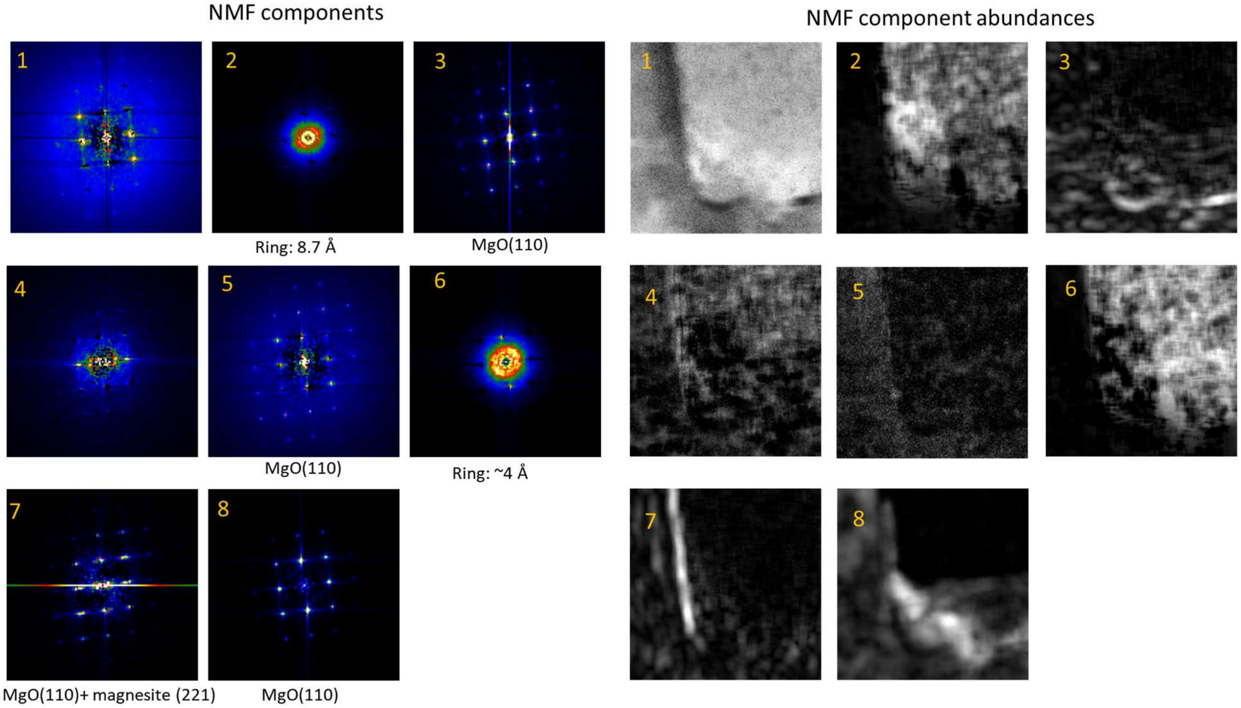

Fig. S6. NMF analysis of Figure 5c.

NMF analysis was conducted using 8 components: for this number of components, each produces a distinct spatial distribution pattern. The layer growth experiments were performed on the (100) MgO substrate. The results are dominated by the periclase, as evident from the “average” component 1 that mostly looks like MgO (100), and visible periclase reflections or shadows thereof in all other components. Components 3, 5, and 8 are periclase MgO (100) patterns with varying degrees of diffuseness, with Component 8 appearing the sharpest. The remaining components show non-periclase contributions: Component 2 is an amorphous

ring pattern of for 8.7 Å lattice spacing and Component 6 an amorphous ring pattern for 4 Å lattice spacing, both localized in the reaction layer. Component 7 is the only one containing magnesite (221) reflections (by comparison with simulated SAED in CrystalMaker Single Crystal); it is mostly localized in a thin layer at the boundary of the reaction area.

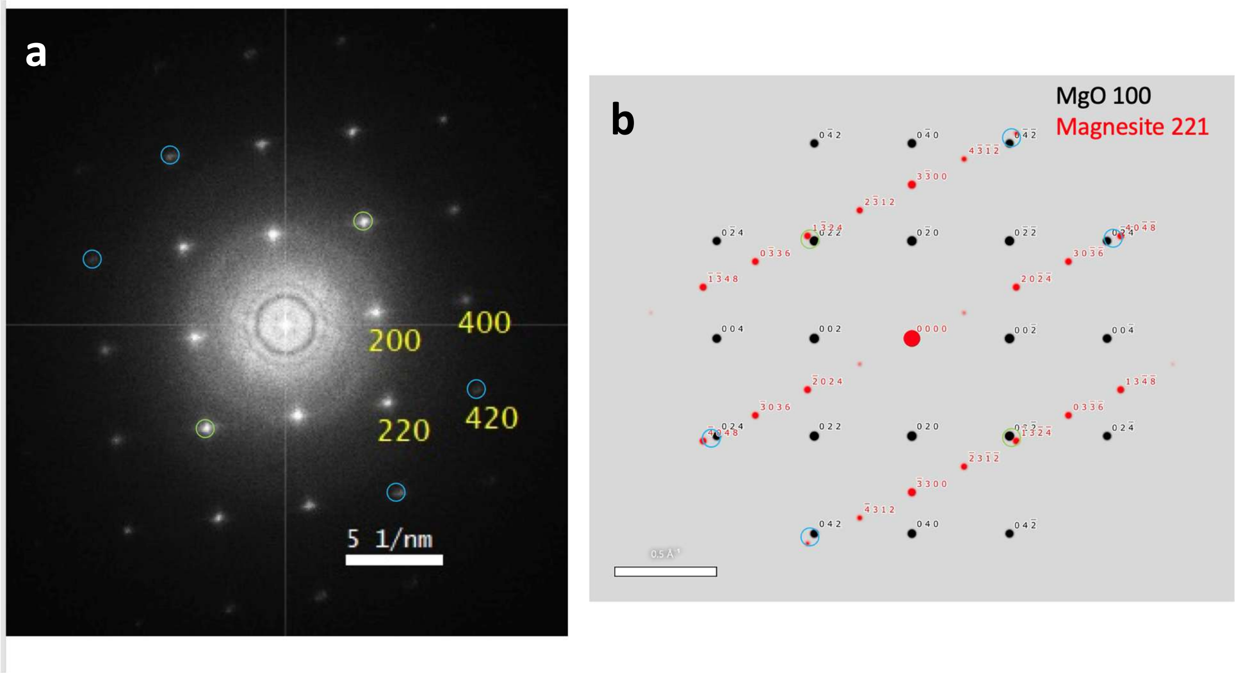

Fig. S7. Identification of crystalline phases in Figure 5c using a diffractogram. a) Diffractogram of Figure 5c. b) Overlay of SAED for MgO(001) and magnesite (221) simulated with CrystalMaker Single Crystal. The equivalent split reflections are highlighted by the blue circles in both (a) and (b). Note that the experimental diffractogram and the simulated diffraction pattern are approximately mirror images of each other; both scale bars are 5 nm^{-1}.

4. Results: Effects of Humidity Over Longer Time Periods

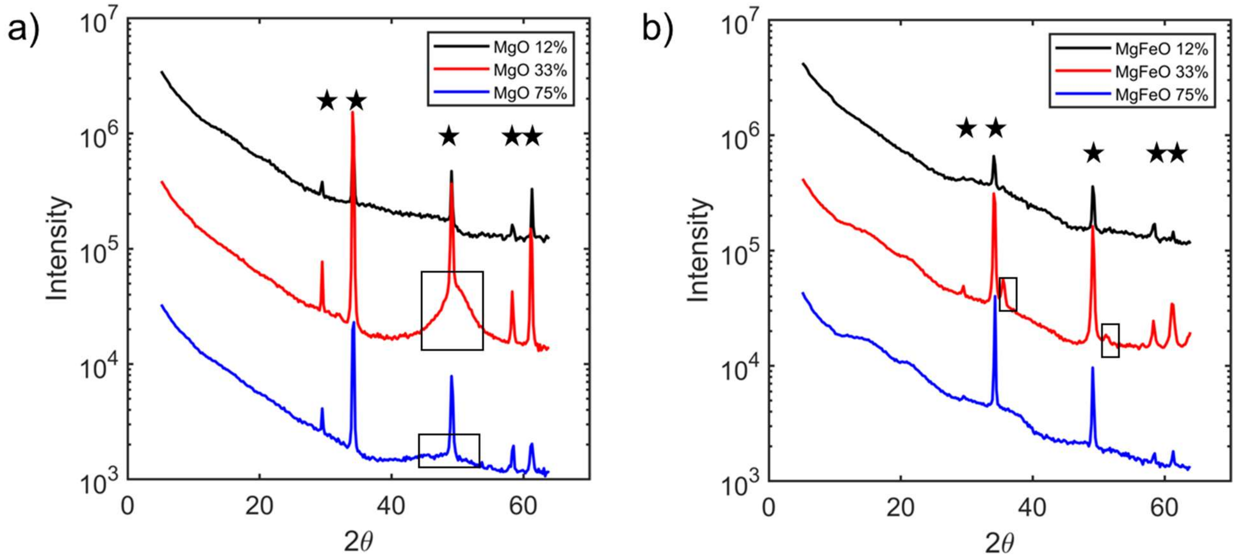

Fig. S8. GIXRD measurements from (a) MgO[2] and (b) (Mg,Fe)O samples exposed to air at 33% R.H and 75% R.H for 8 days. Stars represent the location of periclase Bragg peaks due to penetration of X-rays into the surface. Boxes represent features that may be due to secondary phase formation. MgO data adapted from reference [2]. Copyright 2024 American Chemical Society.

Table S5. XRR fit results, film density (ρ), thickness (d), roughness (σ) and goodness of fit (χ2), for (Mg,Fe)O samples reacted in air at 33% and 75% relative humidity for 8 days.

R.H., time	layer 2			layer 1			Substrate	χ2
	ρ (g/cm^3) (±)	d (Å) (±)	σ (Å) (±)	ρ (g/cm^3) (±)	d (Å) (±)	σ (Å) (±)	σ (Å) (±)	
33%, 8 Days	0.20<br>0.03	7.7<br>0.2	1.8<br>0.3	1.35<br>0.01	17.2<br>0.1	3.4<br>0.1	1.60<br>0.01	4.56
75%, 8 Days	0.86<br>0.01	24.9<br>0.3	5.05<br>0.03	2.41<br>0.01	3.9<br>0.3	7.1<br>0.1	1.35<br>0.02	5.25
75%, 8 Days (Sample 2)	1.06<br>0.03	10.3<br>0.2	4.6<br>0.1	0.73<br>0.02	17.2<br>0.1	3.1<br>0.1	2.50<br>0.01	7.05

5. Results: Effects of CO_2

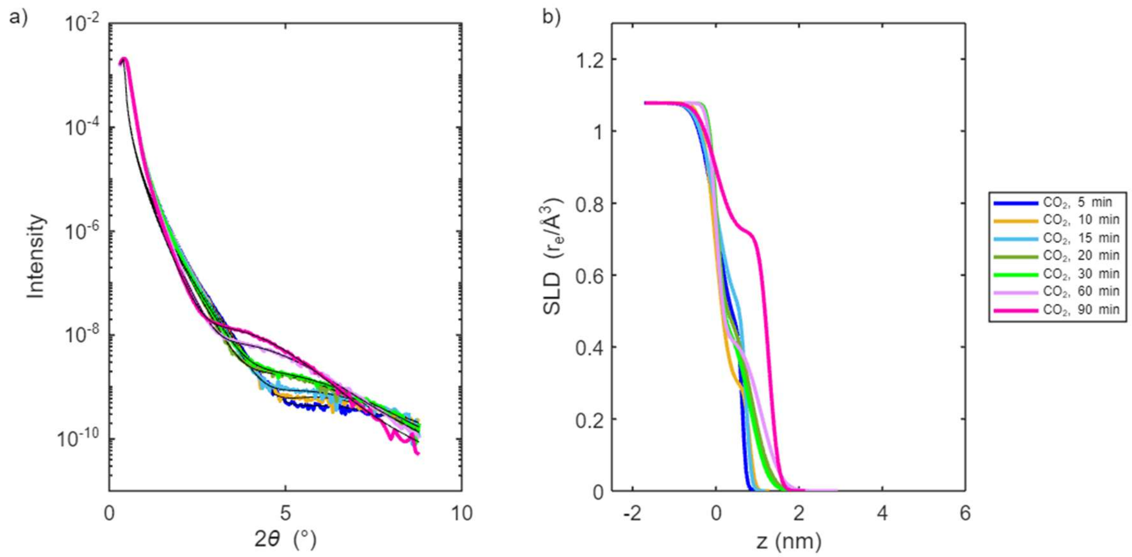

Fig. S9. (a) X-ray reflectivity measurements and fits (black lines) and scattering length density profiles from the fits for (Mg,Fe)O samples reacted in humid CO_2 for 5-90 minutes.

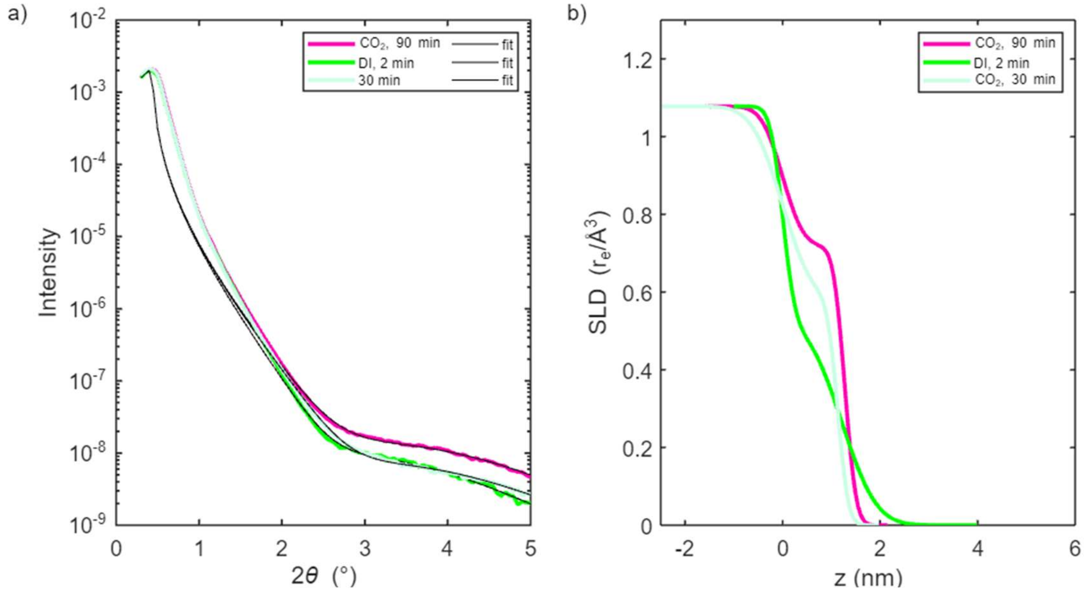

Fig. S10. (a) X-ray reflectivity measurements and fits (black lines) and scattering length density profiles from the fits for (Mg,Fe)O samples reacted in humid CO_2 for 90 minutes, followed by DI water, followed by humid CO_2 for 30 minutes.

Table S6. XRR fit results, film density (ρ), thickness (d), roughness (σ) and goodness of fit ($\chi 2$), for (Mg,Fe)O sample reacted in humid CO_2 and deionized water for various amounts of time.

R.H., time	layer 1			Substrate	$\chi 2$
	ρ (g/cm^3) (±)	d (Å) (±)	σ (Å) (±)	σ (Å) (±)	
>95%, 5 min CO_2, 10 Min Dry N_2	1.36<br>0.08	6.41<br>0.08	0.7<br>0.2	3.44<br>0.08	9.97
>95%, 10 min CO_2, 10 Min Dry N_2	0.96<br>0.01	8.09<br>0.02	1 (Fixed)	2.53<br>0.01	11.63
>95%, 15 min CO_2, 10 Min Dry N_2	1.63<br>0.05	7.27<br>0.05	0.97<br>0.07	3.41<br>0.07	12.03
>95%, 20 min CO_2, 10 Min Dry N_2	1.74<br>0.04	8.49<br>0.04	3.67<br>0.06	1.39<br>0.04	11.65
>95%, 30 min CO_2, 10 Min Dry N_2	1.57<br>0.03	8.23<br>0.04	3.34<br>0.06	1.41<br>0.04	8.19
>95%, 60 min CO_2, 10 Min Dry N_2	1.42<br>0.01	10.93<br>0.02	3.69<br>0.03	1.54<br>0.01	10.24
>95%, 90 min CO_2, 10 Min Dry N_2	2.38<br>0.01	12.75<br>0.02	1.83<br>0.01	3.41<br>0.03	9.77
2 min DI water, 10 Min Dry N_2	1.76<br>0.02	12.17<br>0.03	5.60<br>0.05	2.01<br>0.02	12.30
>95%, 30 min CO_2, 10 Min Dry N_2	1.90<br>0.01	11.34<br>0.03	1.71<br>0.02	4.96<br>0.04	9.14

6. Results: Effects of CO_2 on Longer Time Periods

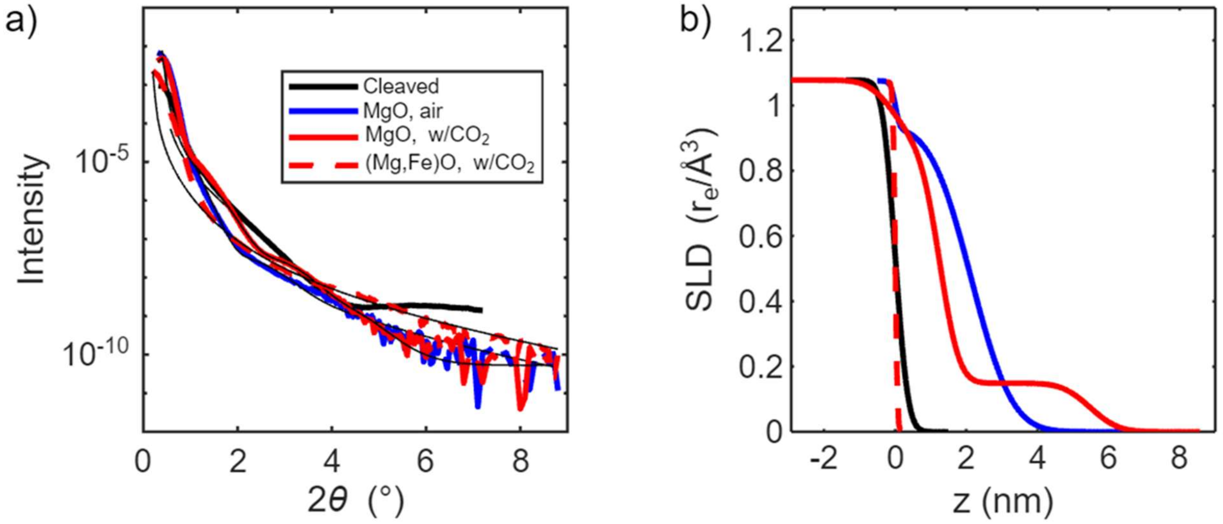

Fig. S11. XRR profile (a) of MgO and (Mg,Fe)O samples reacted under 33% R.H. in air or CO_2 for 30 days and their scattering length density (SLD) profiles (b) from the fits of the data. MgO data adapted from reference [1]. Copyright 2025 American Chemical Society.

Table S7. XRR fit results, film density (ρ), thickness (d), roughness (σ) and goodness of fit (χ2), for (Mg,Fe)O sample reacted for 30 days in air at 33% and 75% humidity in air or CO_2.

R.H., time	layer 2			layer 1			Substrate	χ2
	ρ (g/cm^3) (±)	d (Å) (±)	σ (Å) (±)	ρ (g/cm^3) (±)	d (Å) (±)	σ (Å) (±)	σ (Å) (±)	
(Mg,Fe)O 33%, 30 Days w/CO_2				0<br>(Fixed)	0<br>(Fixed)	0.5<br>0.5	0.5<br>0.5	2.88
(Mg,Fe)O 75%, 30 Days w/CO_2	1.05<br>0.01	16.40<br>0.06	4.35<br>0.04	1.48<br>0.01	38.76<br>0.04	2.05<br>0.07	1.85<br>0.01	33.00
(Mg,Fe)O 75%, 30 Days	1.10<br>0.02	15.9<br>0.2	4.67<br>0.07	1.59<br>0.03	18.4<br>0.1	3.5<br>0.2	1.77<br>0.02	5.50

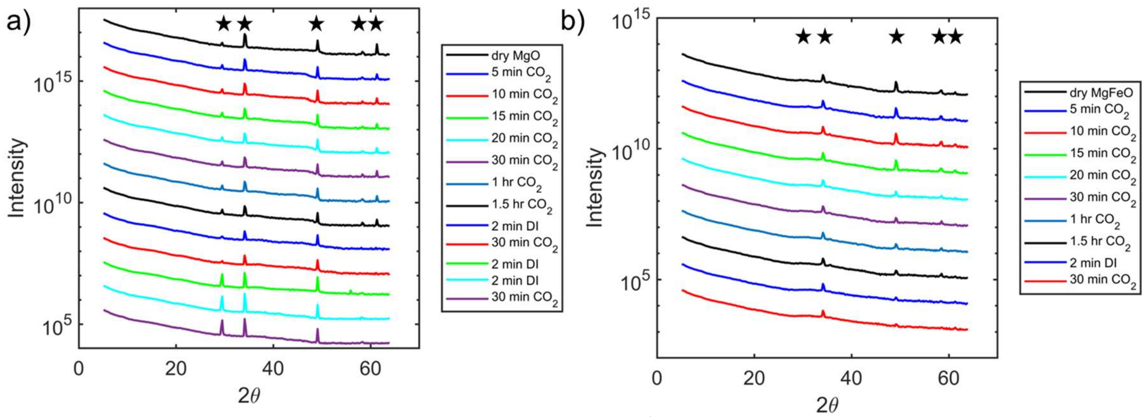

Fig. S12. GIXRD measurements from the (a) MgO[1] and (b) (Mg,Fe)O samples exposed to humid CO_2 and deionized water for varying amounts of time. Stars represent the location of periclase Bragg peaks due to penetration of X-rays into the topmost layers of the substrate. MgO data reproduced from reference [1]. Copyright 2025 American Chemical Society.

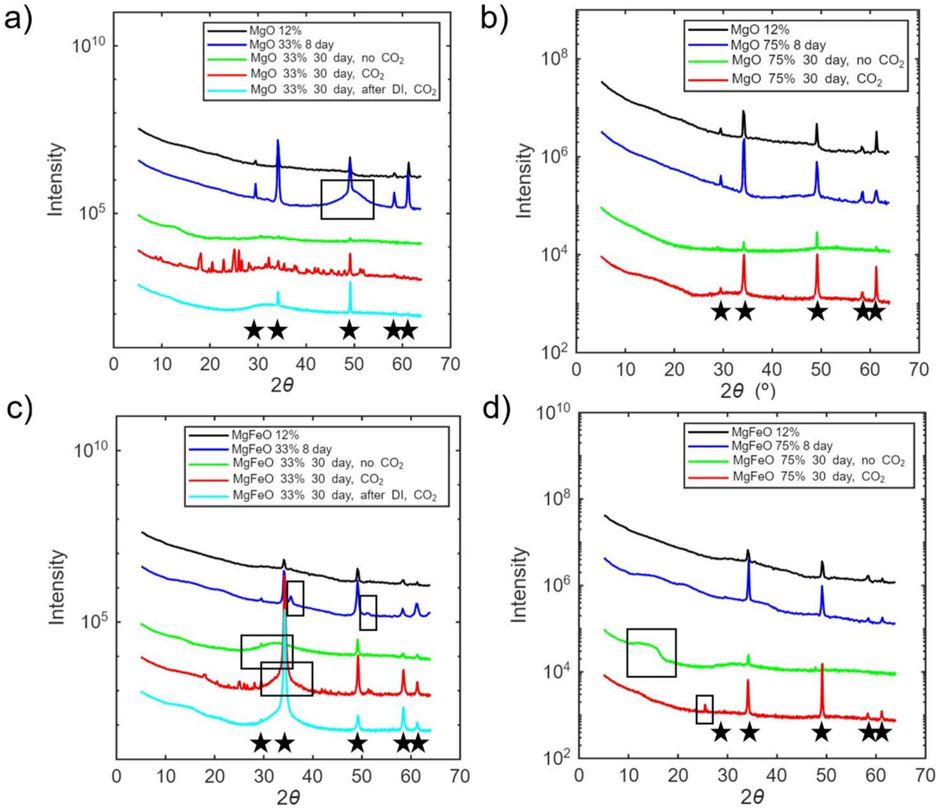

Fig. S13. GIXRD measurements from MgO[1] samples post cleaving (12% relative humidity and to air at (a) 33% R.H and (b) 75% R.H for 30 days in air or CO_2 and (Mg,Fe)O samples post cleaving (12% relative humidity and to air at (c) 33% R.H and (d) 75% R.H for 30 days in air or CO_2. Stars represent the location of periclase Bragg peaks due to penetration of X-rays into the surface. The MgO and (Mg,Fe)O samples reacted at 33% humidity in CO_2 have many peaks present. For the other measurements, boxes represent features that may be due to secondary phase formation. MgO data reproduced from reference [1]. Copyright 2025 American Chemical Society.

References

(1) Yang, P.; Bracco, J. N.; Meneses, G. C.; Yuan, K.; Stubbs, J. E.; Boamah, M.; Sassi, M.; Eng, P. J.; Boebinger, M. G.; Borisevich, A.; et al. Carbonation of MgO Single Crystals: Implications for Direct Air Capture of CO2. *Environmental Science & Technology* **2025**. DOI: 10.1021/acs.est.4c09713

(2) Bracco, J. N.; Camacho Meneses, G.; Colón, O.; Yuan, K.; Stubbs, J. E.; Eng, P. J.; Wanhala, A. K.; Einkauf, J. D.; Boebinger, M. G.; Stack, A. G.; et al. Reaction Layer Formation on MgO in the Presence of Humidity. *ACS Applied Materials & Interfaces* **2024**, *16* (1), 712-722. DOI: 10.1021/acsami.3c14823.
